# Increased T Cell Differentiation and Cytolytic Function in Bangladeshi Compared to American Children

**DOI:** 10.3389/fimmu.2019.02239

**Published:** 2019-09-20

**Authors:** Lisa E. Wagar, Christopher R. Bolen, Natalia Sigal, Cesar J. Lopez Angel, Leying Guan, Beth D. Kirkpatrick, Rashidul Haque, Robert J. Tibshirani, Julie Parsonnet, William A. Petri, Mark M. Davis

**Affiliations:** ^1^Department of Microbiology and Immunology, Stanford University, Stanford, CA, United States; ^2^Institute for Immunity, Transplantation, and Infection, Stanford University, Stanford, CA, United States; ^3^Data Sciences and Statistics, Stanford University, Stanford, CA, United States; ^4^Department of Microbiology and Molecular Genetics, University of Vermont College of Medicine and Vaccine Testing Center, Burlington, VT, United States; ^5^International Centre for Diarrhoeal Disease Research, Bangladesh, Dhaka, Bangladesh; ^6^Departments of Medicine and of Health Research and Policy, Stanford University, Stanford, CA, United States; ^7^Department of Medicine, Pathology, and Microbiology, Immunology and Cancer Biology, University of Virginia, Charlottesville, VA, United States; ^8^Howard Hughes Medical Institute, Stanford University School of Medicine, Stanford, CA, United States

**Keywords:** pediatric immunity, CyTOF, environment, immune development, immune profiling

## Abstract

During the first 5 years of life, children are especially vulnerable to infection-related morbidity and mortality. Conversely, the Hygiene Hypothesis suggests that a lack of exposure to infectious agents early in life could explain the increasing incidence of allergies and autoimmunity in high-income countries. Understanding these phenomena, however, is hampered by a lack of comprehensive, direct immune monitoring in children with differing degrees of microbial exposure. Using mass cytometry, we provide an in-depth profile of the peripheral blood mononuclear cells (PBMCs) of children in regions at the extremes of exposure: the San Francisco Bay Area, USA and an economically poor district of Dhaka, Bangladesh. Despite variability in clinical health, functional characteristics of PBMCs were similar in Bangladeshi and American children at 1 year of age. However, by 2–3 years of age, Bangladeshi children's immune cells often demonstrated altered activation and cytokine production profiles upon stimulation with PMA-ionomycin, with an overall immune trajectory more in line with American adults. Conversely, immune responses in children from the US remained steady. Using principal component analysis, donor location, ethnic background, and cytomegalovirus infection status were found to account for some of the variation identified among samples. Within Bangladeshi 1-year-olds, stunting (as measured by height-for-age z-scores) was found to be associated with IL-8 and TGFβ expression in PMA-ionomycin stimulated samples. Combined, these findings provide important insights into the immune systems of children in high vs. low microbial exposure environments and suggest an important role for IL-8 and TGFβ in mitigating the microbial challenges faced by the Bangladeshi children.

## Introduction

Infants and young children are particularly vulnerable to infectious diseases (1–3). Before the widespread availability of vaccines, ~40% of children died before reaching 5 years old, mostly due to infections (4, 5). Although vaccines and modern medicine have mitigated this vulnerability significantly, in poorer areas of the world where pathogen exposure is elevated, many children are at risk for either infection-related mortality or, more commonly, lifelong disability such as stunting and poor cognitive development. This latter condition is often accompanied by environmental enteropathy (EE), a subclinical condition common in developing countries, characterized by intestinal inflammation, lymphocyte infiltration, and damage to the gut epithelium (6–8). Observational and mouse model studies have pointed to repeated enteric pathogen exposure as a potential driver of the condition (9, 10). It is reported that EE contributes to poor nutrient absorption, malnutrition, developmental delay, and potentially oral vaccine underperformance in children (7, 8, 11, 12).

The pediatric immune system undergoes many changes during the first 5 years of life (13–15). It is important to understand differences in pediatric immune responses in high- and low-income countries, as these differences can affect the success of interventional strategies such as immunization (16). For example, oral rotavirus vaccine was remarkably effective in Finland and the United States (98%) but only 50–80% effective in Nicaragua, Malawi, and South Africa (17–19). Numerous studies have investigated human population-level differences in immune responses, with a focus on either genetic or environmental influences. Innate cells from adults and infants have been well-characterized and both genetic- and environment-driven effects appear to play a role in how these responses are shaped (20–27). Investigations of pediatric populations support the idea that these alterations occur early in life (28). The diversity and level of pathogen burden have been shown to broadly alter Th1/Th2 cytokine bias (23, 29, 30). Antigen-specific responses have also been studied for infectious diseases with higher incidence in low-income countries, such as tuberculosis (31, 32). However, relatively little is known about the effect of environment more broadly on the early events of developing adaptive immunity and to our knowledge, no studies have directly compared deeply-profiled peripheral blood mononuclear cells (PBMCs) from children in high vs. low pathogen environments.

It has been proposed that some degree of diverse microbial exposure is beneficial to developing immune systems, tuning them in some way to avoid childhood allergies and early onset autoimmunity; this is known as the “Hygiene Hypothesis,” proposed by Strachan (33). Given the long-term consequences of a highly immunostimulatory environment on early-life innate immunity, we posited that a pathogen-rich setting would profoundly alter adaptive immune development and function early in life.

To gain further insights into how environment influences immunologic development in children, immune profiles of PBMCs from children in two cohorts, Performance of Rotavirus and Oral Polio Vaccines in Developing Countries (PROVIDE) and Stanford's Outcomes Research in Kids (STORK), were examined. The PROVIDE study was designed to explore the effects of environmental enteropathy and enteric infections on development and immunity by tracking infants born in Mirpur, a poor district of Dhaka, Bangladesh from birth through 4 years of age (34). The STORK study tracked childhood development in 1–3 year old children located in the San Francisco Bay Area, USA (35). We analyzed samples from an overlapping age range of 1–3 years of age in both cohorts using time-of-flight mass cytometry (CyTOF) for deep immune profiling with a focus on T cell differentiation and their responses to stimulation. Longitudinal samples were available for a subset of the participants. At the initiation of this study, it was unknown what cell populations would be of interest in dissecting the role of environment on immunity. Thus, we developed a panel that would cover all of the major immune populations found in pediatric PBMCs (mostly T cells, B cells, NK cells, and to a lesser extent, monocytes). In addition, we measured the functional capacity of these cell types with a broad array of cytokines and activation markers. We compared the phenotype and function of unstimulated and *ex vivo* phorbol-12-myristate-13-acetate (PMA)-ionomycin-stimulated PBMCs to determine how environment shapes immune development and function early in life, and whether immune changes correlate with clinical measures of health.

## Results

### Clinical Features of PROVIDE (Bangladeshi) and STORK (American) Cohorts

**Figure 1:**
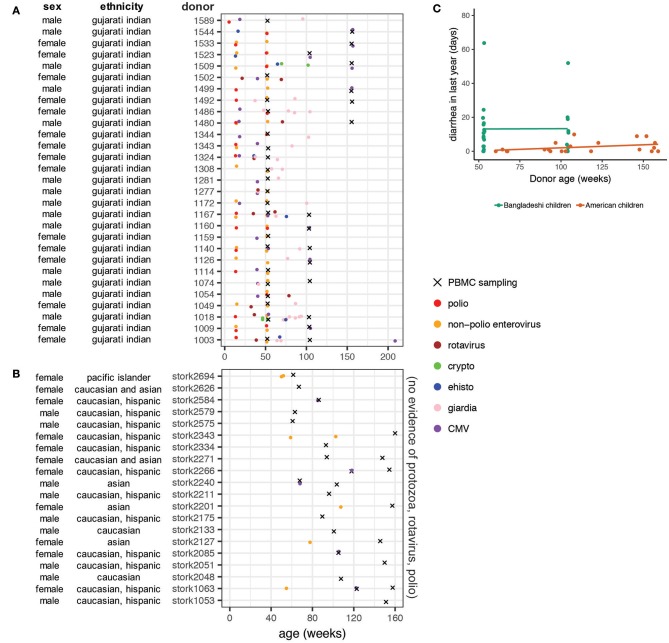
Basic characteristics and clinical data on donor populations. Ethnicity from the PROVIDE cohort is based on genome-wide association study data showing the cohort is genetically homogeneous and most closely resembles the Gujarati Indians from Houston from the HapMap project. Ethnicity data from the STORK cohort is self-reported by the mothers of the infants. **(A)** Stool samples from the Bangladeshi cohort (PROVIDE) were tested for cryptosporidium, giardia, and E histolytica when infants presented with diarrhea and surveilled for shedding of polioviruses (including vaccine strains) and non-polio enteroviruses on weeks 14 and 52. **(B)** PBMC sampling periods for the STORK cohort of American children. Note that no infection data were available for the American children included in the study but based on medical chart review and vaccination status, parasitic, rotavirus, and poliovirus infections are unlikely. **(C)** Diarrheal surveillance data in Bangladeshi and American children. Diarrheal incidence data were available for the first 2 years of life in the Bangladeshi cohort and throughout the entire study period in the American cohort. Rates were found to be significantly different (*p* = 0.015) using a linear mixed effects model that accounts for repeat samples from the same donor.

The PROVIDE cohort subjects were monitored for evidence of enteric infections and diarrheal disease for the first 2 years of life. Stool samples were collected and tested for a panel of infectious agents, including protozoa (*cryptosporidium, Entamoeba histolytica, giardia*) and common enteroviruses (polioviruses, rotavirus, and other non-polio enteroviruses). At least one of these agents was detected in all of the PROVIDE subjects tested in this study (Figure 1A). Non-polio enteroviruses were the most common infection, with 83% of donors testing positive at least once. A third of donors tested rotavirus positive in their stool at least once during the surveillance period. Protozoa were also prevalent, with *giardia* being the most common (59% of participants). The presence of poliovirus is most likely due to immunization with oral polio vaccine or from vaccine-strain polioviruses in the environment. Most donors tested positive for cytomegalovirus (CMV) antibodies, indicating early infection in the Bangladeshi cohort was common. We examined PBMC samples for CyTOF analysis from weeks 53 (*n* = 18 unstimulated; *n* = 19 stimulated), 104 (*n* = 9), and 156 (*n* = 6).

The STORK cohort is an ethnically and socioeconomically diverse cohort of infants living in the San Francisco Bay Area of the United States (Figure 1B). No protozoan infections were detected or suspected in this cohort and the rotavirus vaccine, which has high effectiveness in the United States (36), is part of the standard immunization schedule in California. Waterborne disease outbreaks are not common in California (37, 38). Inactivated polio vaccine is used in the United States, thus poliovirus infections are not likely. Some subjects (25%) were diagnosed with viral gastroenteritis at least once during the study (Figure 1B). A small number (*n* = 5) of the STORK donors tested positive for CMV. Other common diagnoses included upper respiratory infection, rash, fever with no etiology specified, dermatitis, and evaluation for allergy. For analysis purposes, we binned PBMC samples from the STORK cohort to the nearest equivalent sampling period as the PROVIDE cohort: weeks 53 (*n* = 5), 104 (*n* = 11), and 156 (*n* = 8).

Children in the PROVIDE cohort had significantly more diarrheal disease than American children, independent of age (Figure 1C; *p* = 0.015 using a mixed linear effects model to account for repeat donors). The available infection surveillance and diarrheal disease data show that enteropathogen exposure and diversity differ between the Bangladeshi and American cohorts. These findings are consistent with previous studies that found a high enteropathogen burden in children growing up in urban slum regions of Bangladesh (12, 39–42).

### Automated Clustering Effectively Delineates Complex Cell Types With Minimal User Direction

To investigate the high-dimensional CyTOF data in a minimally directed way, we employed a dimensionality reduction algorithm coupled with semi-automated clustering to characterize and simplify the complex expression patterns of 23 (unstimulated) or 32 (PMA-ionomycin) of the parameters measured. We used the t-stochastic neighborhood embedding (tSNE) technique utilized in other CyTOF applications to reduce the dimensionality of our data on unstimulated and PMA-ionomycin stimulated samples as separate analyses. Like other tSNE-based algorithms (43–46), cells with similar marker expression patterns cluster more closely in 2-dimensional space and the data can be visualized with a clustering map. Then, using a custom designed clustering algorithm, referred to as AdjClust hereafter, we defined groups of similar cells based on both the 2-dimensional maps and the original *N*-dimensional dataset. AdjClust allows the user to manually assign relative weighting to the markers to be used in the clustering, thus providing more control over which markers are considered useful for clustering and characterization. We performed a preliminary test of the clustering with a mixture of unstimulated and PMA-ionomycin stimulated cells. AdjClust appropriately separated cells with distinctive activation and functional profiles into different clusters (Supplementary Figure 1).

### Increased Effector and Senescent-Phenotype T Cells in Bangladeshi 2- and 3-Year-Olds

**Figure 2:**
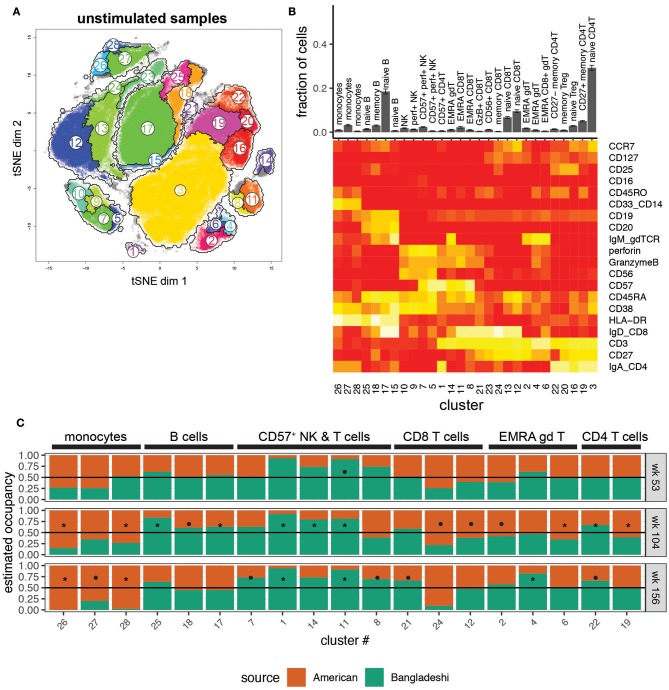
Location-based differences in immunophenotypes identified with automated clustering. Markers used for the tSNE and clustering processes were selected based on relevence to cell identification under unstimulated conditions. Samples include 24 from American children (week 53 *n* = 5, week 104 *n* = 11, week 156 *n* = 8), 33 from Bangladeshi children (week 53 *n* = 18, week 104 *n* = 9, week 156 *n* = 6), and 30,000 randomly sampled cells from 3 American adults. **(A)** tSNE map with overlaid AdjClust clusters on aggregate of all samples included in the analysis. Using AdjClust, 28 unique clusters were identified that delineated cell phenotypes of interest. Minimum cluster size was set to 1,500 cells. **(B)** Distribution of cells among AdjClust clusters in unstimulated samples from combined Bangladeshi and American children's PBMC samples and their phenotypic characteristics. Values plotted are means ± SEM. **(C)** Ratio of cluster occupancy between age-matched Bangladeshi vs. American children. Only clusters with FDR < 0.01 are shown. For each time point, Mann-Whitney tests were performed for each of the 28 clusters. *p*-values were then corrected for multiple testing using the Benjamini and Hochberg method. Significance levels of the cluster occupancy differences between Bangladeshi and American children are indicated by FDR < 0.1 (•), FDR < 0.05 (*).

We first investigated the phenotypes of unstimulated PBMCs from Bangladeshi and American children. We identified 28 clusters (see Supplementary Table 1 for cluster assignment quick-reference) that accurately separated biologically meaningful populations and reflected their diverse phenotypes (Figure 2A). Even small populations, such as effector memory RA (EMRA) and CD57^+^ CD4, CD8, and γδ T cells were effectively clustered using AdjClust (Figure 2B). We stratified the data by donor age and study location to investigate cohort-based differences in immune phenotypes. A “cluster occupancy” value was determined for each sample; the occupancy value refers to the fraction of total cells from a sample that falls within a given cluster. We then calculated the relative contribution (“estimated occupancy”) of cells coming from age-matched Bangladeshi and American children's samples to understand differences in the distribution of their cell types.

There were surprisingly few significant differences in immune phenotypes in Bangladeshi vs. American 1-year-olds (Figure 2C; Mann-Whitney test with BH correction; FDR = 0.07 for cluster 11, EMRA CD8 T cells). However, there were several trends indicating differences between the two locations at 1 year of age that became more robust with increasing age. Bangladeshi 2-year-olds had fewer monocytes (FDR = 0.03 and 0.04; clusters 26 and 28), naive CD8 T cells (FDR = 0.07; cluster 12), and early-differentiated (i.e., CD27^+^) memory CD4 T cells (FDR = 0.02; cluster 19) relative to American children. They also showed a decrease in three small populations of EMRA phenotype T cells (CD8 T cells FDR = 0.07, cluster 24; γδ T cells FDR = 0.04 and 0.08, clusters 6 and 2). Bangladeshi 2-year-olds had increased naive and memory B cells (FDR = 0.03, 0.04, and 0.09; clusters 25, 17, and 18) and highly differentiated T cells (CD57^+^ CD4 T cells FDR = 0.02, cluster 1; EMRA γδ T cells FDR = 0.02, cluster 14; EMRA CD8 T cells FDR = 0.03, cluster 11; CD27^−^ CD4 T cells FDR = 0.03, cluster 22). The reduced monocyte (FDR = 0.03, 0.05, 0.03; clusters 26, 27, 28) and increased effector and cytolytic phenotypes (NK cells FDR = 0.06, cluster 7; CD8 T cells FDR = 0.03, 0.05, 0.06, clusters 11, 8, 21; γδ T cells FDR = 0.05, cluster 4; CD4 T cells FDR = 0.05, 0.08, clusters 1, 22) also persisted in Bangladeshi 3-year-olds. Overall, these findings suggest that Bangladeshi children have fewer peripherally-circulating monocytes and more differentiated T cell subsets compared to American children and that these effects become more pronounced with increasing age.

**Figure 3:**
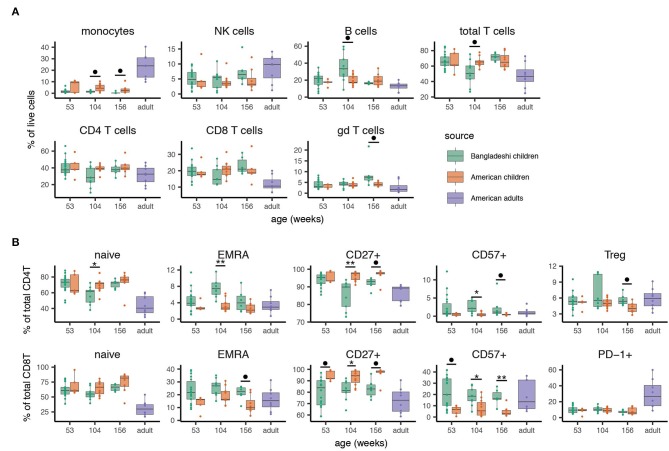
Manual gating validation of altered immune phenotypes associated with location. After identifying cell subsets that differed between Bangladeshi and American cohorts with automated analyses, main cell populations and phenotypes of interest were manually gated. Samples include 24 from American children (week 53 *n* = 5, week 104 *n* = 11, week 156 *n* = 8) and 33 from Bangladeshi children (week 53 *n* = 18, week 104 *n* = 9, week 156 *n* = 6). Cell phenotypes from 8 American adults are shown as a reference for typical values derived from the CyTOF panel and gating scheme utilized here and were not used for statistical analyses. **(A)** Minor differences in main cell populations are detectable between equivalent-aged Bangladeshi and American children. Bangladeshi children show reduced monocyte and total T cell populations and increased B cell and gamma delta T cell populations compared to American children at select ages. **(B)** Bangladeshi children show more T cell differentiation and senescence phenotypes but not exhaustion compared to equivalent-aged American children. To compare Bangladeshi and American children's cell populations from manual gating, Mann-Whitney tests were performed for each population and time point shown. *p*-values were then corrected for multiple testing using the Benjamini and Hochberg method. Significance levels of the cell population differences between Bangladeshi and American children are indicated by FDR < 0.1 (•), FDR < 0.05 (*), FDR < 0.01 (**). Values plotted are means ± SEM.

We then validated the automated clustering findings using manual gating with standard markers for main cell populations and T cell subsets (see Supplementary Figure 2 for representative gating). We confirmed via manual gating that monocytes (and transiently at age two, total T cells) were reduced among Bangladeshi children while total B cells and γδ T cells were elevated at select time points (Figure 3A; significance determined by Mann-Whitney with BH correction for multiple testing). These findings closely matched those identified using AdjClust. Given the strong signal for T cell differentiation in the automated analysis, we further investigated the frequency of naive, central memory, effector memory, and EMRA T cell phenotypes (using CD45RA, CD45RO, and CCR7) and other differentiation indicators (CD27, CD57, PD-1, regulatory T cells). Compared to age-matched American children, Bangladeshi children's CD4 and CD8 T cells were indeed more differentiated (Figure 3B). At many time points, Bangladeshi children had fewer CD27^+^ T cells and more CD57^+^ cells, suggesting differentiation toward a more effector-like and potentially senescent (47) phenotype. In nearly all instances where Bangladeshi children's T cells differed from those of American children, they more closely resembled an adult phenotype. Based on these findings, we determined that the T cells of Bangladeshi children, though more similar to their American counterparts at 1 year of age, are more differentiated at ages two and three and increasingly resemble the proportions of differentiated T cells from adults.

### Cytokine Suppression and Shift to Cytotoxicity in T Cells From Bangladeshi 2-Year-Olds

**Figure 4:**
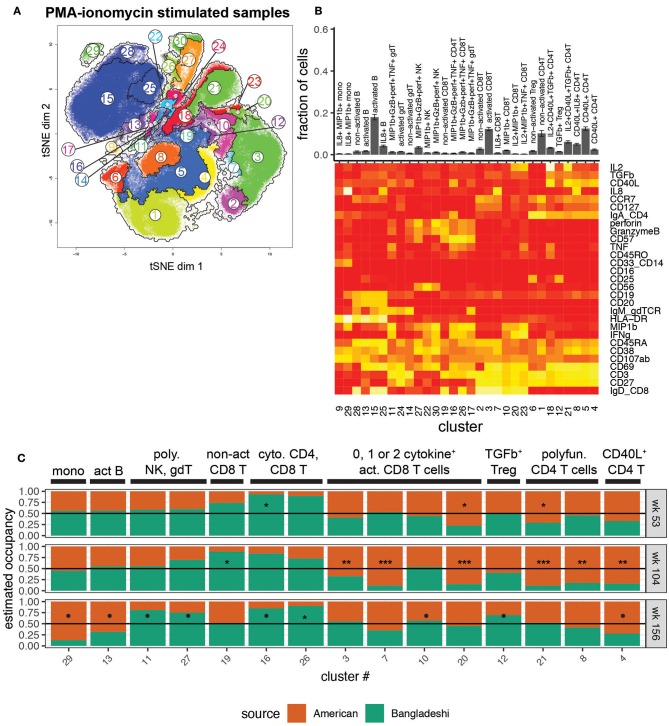
Location-based differences in functional responses to stimulation. Markers used for the tSNE and clustering processes were selected based on relevance to cell phenotypes and functional characterization. Samples include 24 from American children (week 53 *n* = 5, week 104 *n* = 11, week 156 *n* = 8), 34 from Bangladeshi children (week 53 *n* = 19, week 104 *n* = 9, week 156 *n* = 6), and 30,000 randomly sampled cells from 3 American adults. **(A)** tSNE map with overlaid AdjClust clusters on aggregate of all samples included in the analysis. Using AdjClust, 30 unique clusters were identified that delineated cell differentiation and function/activation. Minimum cluster size was set to 1,500 cells. **(B)** Cell phenotypes and functions identified with AdjClust on PMA-ionomycin stimulated samples. Labels were manually assigned based on marker expression. The fraction of cells in each cluster represents aggregate data from Bangladeshi and American children's stimulated samples. **(C)** Ratio of cluster occupancy between age-matched Bangladeshi vs. American children. Only clusters with FDR < 0.01 are shown. For each time point, Mann-Whitney tests were performed for each of the 28 clusters. *p*-values were then corrected for multiple testing using the Benjamini and Hochberg method. Significance levels of the cluster occupancy differences between Bangladeshi and American children are indicated by FDR < 0.1 (•), FDR < 0.05 (*), FDR < 0.01 (**), FDR < 0.001(***).

To investigate the functional potential of immune cells from the two cohorts, we studied the clustered cell populations from PMA-ionomycin stimulated samples (Figures 4A,B). Thirty clusters of immune cells at varying levels of activation, based on expression of activation markers in combination with zero to four or more cytokines, were created using AdjClust. Similar to the unstimulated cell population analysis, Bangladeshi and American children's stimulated PBMC samples had similar functional profiles at 1 year of age (Figure 4C). Bangladeshi 1-year-olds had a small but detectable population of cytolytic (MIP-1β^+^ granzyme B^+^ perforin^+^ TNF^+^) CD4 T cells that were virtually absent in American children (FDR = 0.01; cluster 16). They also showed a reduced frequency of a large population of CD4 T cells that express CD40L and produce both IL-2 and TGFβ (FDR = 0.04; cluster 21).

At age two, Bangladeshi children's T cells appeared to be broadly defective in their ability to respond to stimulation, with significant increases in non-activated CD8 T cells (FDR = 0.02; cluster 19) and large decreases in numerous activated and cytokine-producing CD4 and CD8 T cells (FDR < 0.001 for all three significant activated CD8 T cell clusters, 3, 7, and 20; FDR < 0.001 for all three significant activated CD4 T cell clusters, 21, 8, 4) compared to American children. Interestingly, by age three this defective response to stimulation was replaced with a shift toward a cytotoxic profile. Compared to American 3-year-olds, Bangladeshi children's γδ, NK, CD4, and CD8 T cells were more likely to produce cytokines such as perforin, granzyme B, TNF, and MIP-1β (FDR = 0.07, 0.08, 0.07, and 0.02, respectively; clusters 11, 27, 16, and 26), in response to stimulation. Bangladeshi children also had an increased population of TGFβ-producing Tregs (FDR = 0.1). Overall, this automated analysis showed that there are a small number of highly active cytolytic (i.e., perforin, granzyme, TNF, MIP-1β) cells in many subsets (NK, γδ T, CD8 T, and even CD4 T cells) in Bangladeshi kids and fewer cells with CD40L, IL-8, and IL-2 phenotypes across many T cell subsets, particularly in 2-year-olds and somewhat at 3 years.

Due to the number of cytokines and activation markers included in this CyTOF panel, manual gating for every functional combination was not practical. Therefore, we focused on a few cytokines that were of interest either for their broad immune cell relevance (IL-8, TGFβ) or identified across multiple populations from the clustering analysis (perforin, granzyme B, MIP-1β). The manual gating validated the findings from AdjClust, with overall increased cytolytic functions in Bangladeshi compared to American children at all ages in a number of T cell and NK cell subsets and suppressed T cell cytokine production (IL-8, TGFβ) at 2 years of age (Supplementary Figure 3). Combined, automated and manual analyses of stimulated PBMC samples point to a significant but transient reduction in the ability of lymphocytes to respond to stimulation (IL-8, TGFβ) in Bangladeshi 2-year-old children and overall development of a cytotoxic (perforin, granzyme B, TNF) functional profile.

### Location Is the Strongest Demographic Feature of Immune Variance

**Figure 5:**
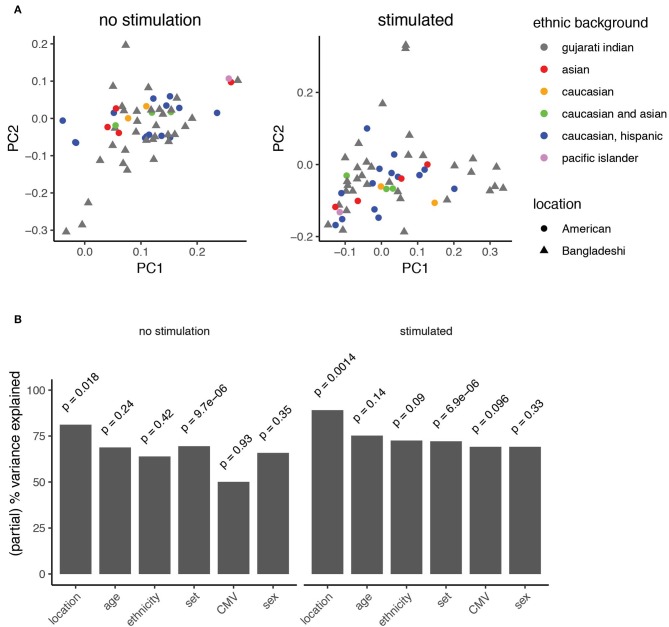
Principal component analysis identifies features explaining immune variation in PBMCs from children. **(A)** Principal component analysis of automated clustering output from PBMCs either left unstimulated (left) or stimulated with PMA-ionomycin (right). Point shapes represent data from American (circles) and Bangladeshi (triangle) cohorts. Points are filled based on reported ethnic background. **(B)** Variance explained by known clinical features. A mixed linear model was used to describe the partial variance explained in PBMC samples from unstimulated (left) and stimulated (right) analyses. Partial percent variance explained (partial eta^2^) calculates the percent variance explained for each variable from the Pillai statistic. A type-II MANOVA test was performed on the linear model and *p*-values for the contribution of each variable were calculated from the Pillai test statistic.

The manual gating analyses (Figure 3, Supplementary Figure 3) suggested that American children's immune profiles were generally more stable throughout the time period studied here, while more variance was observed among Bangladeshi children. The primary goal of this study was to identify environmental, clinical, or other factors that might contribute to pediatric immune development. To accomplish this in an unbiased way, we applied principal component analysis to the AdjClust cluster frequencies from the unstimulated and PMA-ionomycin stimulated analyses (Figure 5A). The principal components were then modeled as a function of available demographic variables (donor location, age, sex, CMV status, and ethnicity) using a linear multivariate multiple regression model. In this study, location serves as a proxy for differences in immune challenges, such as the disparity in exposure to non-polio enteroviruses and CMV (Figure 1). See Supplementary Figure 4 for plots comparing each of the first four principal components against one another. After accounting for batch effects (“set”), we found the strongest biological source of variation out of the features examined was donor location (Figure 5B; partial variance explained 81 and 89% for unstimulated and stimulated samples, respectively; *p* = 0.018 and 0.0014, mixed linear model, type II MANOVA test with *p*-values from Pillai statistic). No other feature tested in the PCA significantly accounted for variance detected in unstimulated cells. In PMA-ionomycin stimulated samples, ethnicity and CMV seroconversion status explained a small amount of the variation but were not strongly significant (73 and 69%, respectively; *p* = 0.09 and 0.096). These data suggest that location, and to a lesser extent, ethnic background and specific infections such as CMV, contribute to immune cell function during pediatric immune development.

### Functional Responses to Stimulation Are Associated With Clinical Stunting in Bangladeshi Children

**Figure 6:**
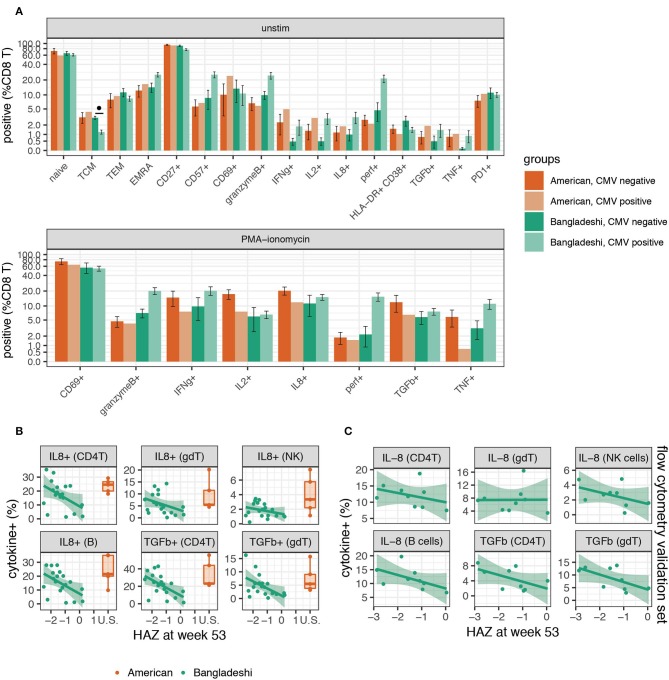
Higher functional responses to stimulation are associated with stunting in Bangladeshi 1-year-olds. **(A)** Frequencies of CD8 T cells with and without stimulation in CMV positive and negative 1-year-olds. Mann-Whitney tests were performed for each population shown. *p*-values were then corrected for multiple testing using the Benjamini and Hochberg method. Significant differences in cell population frequencies between Bangladeshi CMV positive and negative subjects are indicated by FDR < 0.1 (•). Values plotted are means ± SEM. The number of donors in each group were *n* = 4 and 14 Bangladeshi CMV negative and positive, respectively, in unstimulated samples; *n* = 3 and 16 Bangladeshi CMV negative and positive, respectively, in stimulated samples; *n* = 4 American CMV negative for unstimulated and stimulated samples; *n* = 1 CMV positive American child for unstimulated and stimulated samples. **(B)** Relationship between stunting (as measured by height for age z-score, HAZ) and IL-8 or TGFβ cytokine production in unstimulated or PMA-ionomycin stimulated PBMCs from Bangladeshi 1-year-olds (*n* = 8 less stunted; *n* = 8 more stunted). After permuting HAZ scores 5,000 times and setting a cut-off of *p* = 0.05 and FDR = 0.2, we identified IL-8 and TGFβ in stimulated PBMCs as significantly associated with HAZ across multiple populations. No significance was found in unstimulated populations. After PCA and a validation **(C)** set analysis (*n* = 9**)**, the combined *p*-values from training and validation sets were *p* = 0.009 (IL-8) and *p* = 0.002 (TGFβ) using the first principal component. Values from American children (*n* = 5) are presented for comparison purposes.

Given the observation that immune cells from children in Bangladesh were undergoing substantial changes over time, we considered whether immune functions correlated with clinical surrogates of infection and overall health. CMV infection is known to shape much of the immune system (48), particularly CD8 T cells, over time (49) and PCA suggested that CMV infection may explain some of the functional changes observed. Therefore, we asked whether there were any phenotypic or functional differences between Bangladeshi CMV negative (*n* = 4 for unstimulated; *n* = 3 for stimulated) and CMV positive (*n* = 14 for unstimulated; *n* = 16 for stimulated) 1-year-olds' CD8 T cells. Though there was an overall trend toward more differentiation and effector-like functions in CMV positive donors, the sample size for CMV negative Bangladeshi subjects was too small to achieve statistical significance for any particular variable (Figure 6A; statistical analysis by Mann-Whitney test with BH correction). Only a single donor from the STORK cohort was CMV positive at 1 year of age (*n* = 1 CMV positive; *n* = 4 CMV negative), therefore this cohort was not analyzed for the effect of CMV on CD8 T cell phenotypes.

As stunting is a major problem in Bangladeshi children, we were particularly interested in any immunological correlates of this in the PROVIDE cohort, as defined by the height-for-age *z* score (HAZ). Here we identified a chemokine, a cytokine, and an activation marker associated with HAZ score in Bangladeshi 1-year-olds. We performed permutation testing to identify immune cell features that may be involved in the stunting process. Using a cut-off of *p* = 0.05 and FDR = 0.2, we identified seven candidate cell populations that correlated with stunting (statistical analysis details are provided in the methods section). Stunted children made more IL-8 (in B cells, CD4 T cells, γδ T cells, and NK cells) and TGFβ (in CD4 T cells and γδ T cells) after PMA-ionomycin stimulation compared to their less stunted counterparts (Figure 6B). The seventh candidate was a weak association observed for the B cell costimulation molecule CD40L post-stimulation on CD4 T cells. These findings were then validated in a separate set of Bangladeshi 1-year-olds by flow cytometry, where IL-8 (*p* = 0.037; combined *p* = 0.009) and TGFβ (*p* = 0.043; combined *p* = 0.002) were both produced at higher levels in stimulated samples from stunted children (Figure 6C; *n* = 9). Combined, these findings suggest that stunting in Bangladeshi 1-year-olds is correlated with higher functional responses to stimulation, as measured by IL-8 and TGFβ.

## Discussion

The findings of this study indicate that environment plays an important role in shaping the early pediatric immune system. By performing a high dimensional analysis of PBMCs, we found numerous differences in immune function between Bangladeshi and American children at key developmental milestones. Principal component analysis indicated that, of the clinical features we were able to examine, location explained the most variance. This corresponds with what we know about the relative genetic diversity of our cohorts. The American STORK cohort is a more genetically diverse group (Figure 1) yet immunologically similar to one another, whereas the PROVIDE study participants were more genetically homogenous yet immunologically variable. Natural selection events have certainly shaped innate immune-related genes among geographically disparate human populations (20, 27, 50–52), which could have consequences on the subsequent adaptive response. Indeed, we find that ethnic background does explain some of the variance detected among samples stimulated with PMA-ionomycin (Figure 5). However, genetic studies of European and Asian populations, which represent the majority of study participants, find these groups have undergone similar genetic selection events compared to African populations (53–56). This might explain why we see a stronger correlation with location rather than genetic factors. Together, these data suggest that non-genetic influences are strong contributors to the immune variance observed here.

What might these non-genetic influences be? We propose that microbial exposure, maternal health during pregnancy, breastfeeding duration, immunization, chronic infection, hygiene, and diet could all be possible contributors. The socioeconomic disparity between these cohorts also links a number of these factors together, making it difficult to disentangle individual contributors. Though we were unable to consider all non-genetic influences individually in the cohorts described here, we discuss below some of the most likely contributions to the immune variation observed.

The immunization schedules up to 1 year of age are quite similar between the PROVIDE cohort and the standard California vaccine schedule. The main differences are in live vaccines: Bangladeshi children receive BCG vaccine at birth, are immunized against polioviruses with mostly oral polio vaccine in this study, and did not receive varicella vaccine. It is possible that immunization with live vaccines has long-term consequences on immune development, though we did not find any correlation between oral vaccine responses and the immune cell subsets examined in the PROVIDE cohort (data not shown). Hepatitis B, diphtheria, tetanus, pertussis, *Haemophilus influenzae* type B, pneumococcal, measles, and rubella vaccines were similar in number and timing. Therefore, it is unlikely that vaccination schedules alone explain the immune differences observed between the cohorts. However, based on infection surveillance data (Figure 1) as well as literature, Bangladeshi children are exposed to a more diverse enteropathogen environment, experience diarrheal illness more frequently, and most show evidence of environmental enteropathy by 12 weeks (57).

Given these early microbial differences, it was surprising to find Bangladeshi and American 1-year-olds' peripheral immune profiles to be quite similar. Several studies of infants in low- and middle-income countries have shown that innate immunity is shaped early in life and can differ substantially depending on location (21–25, 28). Previous data also suggest that innate responses to stimulation decrease over the first year of life (22). We see an interesting parallel in the adaptive response at 2 years, where lymphocytes appeared to be largely refractory to stimulation in Bangladeshi children. At 1 year of age, some Bangladeshi children's PBMC phenotypes and functional responses look more like the changes observed at 2 years, suggesting some degree of variance in terms of the timing of acquiring these immune changes. It may take more time for early innate immune changes to have functional consequences on adaptive immune cells' phenotypes and functions.

There are other environmental factors that might explain why immune differences among these cohorts increase with increasing age. Passive immunity to some antigens is conferred by maternal and breast milk antibodies early in life. Bangladeshi babies were exclusively breastfed for a median of 142 days and partial breastfeeding continued for at least the first year of life. However, as the children are weaned, begin eating potentially contaminated solid foods, and are increasingly mobile, their exposure to antigen dramatically increases, while passive immunity wanes. These developmental milestones are coincident with the period in which we see the strongest differences between Bangladeshi and American children. Dampened functional responses and enhanced immune maturation, as exhibited by reduced cytokine production and more T cell differentiation in Bangladeshi 2-year-olds (Figure 4), may be a way to protect against pathological inflammatory damage in a high pathogen environment. These data are consistent with the Hygiene Hypothesis, as immune phenotypes of American children remained virtually unchanged throughout the 2-year time course we analyzed, while the Bangladeshi children were much more variable, especially at 1 year. While American children certainly acquire many childhood diseases, the effect these have on their immune system seems negligible compared to the major shifts we see in Bangladeshi children. Perhaps American children later go through a phase of reduced response to stimulation as well when antigen exposure is high (e.g., at daycare or school) and immune memory forms.

The timing and contribution of chronic infections may also play an important role in shaping adaptive immunity. Though we identified human cytomegalovirus infection as a source of immune variation by PCA (Figure 5B), the study was underpowered to identify a specific cell phenotype or function associated with CMV infection. In our cohort of American 1-year-olds, only one subject was CMV positive. In the PROVIDE cohort, early CMV infection was the norm; 83% of participants were CMV positive before 1 year of age. Although it is known that CMV can alter the function of antigen-specific CD4 T cells in children (58), we mostly observed trends (FDR = 0.1 to 0.2) pointing to increased effector T cell phenotypes (granzyme B, perforin, and CD57 expression) in CMV positive individuals. These trends should be validated in future studies via quantification and characterization of CMV-specific T cell subsets. CMV is a unique human virus that has correlates with profound changes in the immune systems of infected organisms, as shown earlier in CD8 T cells in humans and rhesus macaques (59–61). Brodin et al. also showed that monozygotic twins discordant for CMV seropositivity displayed divergences in almost 60% of over two hundred immunological variables (48). Particularly interesting in terms of the biological implications of CMV infection are the data of Furman and colleagues who found that CMV positive young adults had reproducibly superior antibody responses to influenza vaccination compared to CMV negative individuals (62). The implication that CMV infection can boost the immune response of humans was reinforced by the finding in this same report that mice infected by murine CMV were substantially protected from a subsequent influenza infection (62). Taken together these data suggest that for healthy individuals, CMV infection could be beneficial, though the virus is clearly life threatening for immunocompromised individuals. A question from these studies is whether this boost in immunity is only advantageous for individuals in high income countries like the United States. The fact that we see a trend toward more robust CD8 T cell activity in the CMV positive vs. negative children in this study suggests that CMV infection is generally beneficial regardless of the pathogen environment. This could explain why CMV infection is so common across the globe.

We found that stronger IL-8 and TGFβ production across multiple immune cell types was associated with stunting in Bangladeshi 1-year-olds. Even taking false discovery rate into account, it is not likely that IL-8 and TGFβ are false signals since they were found across numerous cell types. Stunting is thought to be a consequence of poor nutrition combined with other causative factors, as malnutrition alone is not sufficient to induce stunting (63) and previous studies have found no clear association with immune cell composition (64). Interestingly, both IL-8 and TGFβ can play important immunological roles that are relevant to a high enteropathogen environment. TGFβ's effects can vary by cell type, differentiation status, and cytokine milieu (65, 66). However, TGFβ production can provide a protective benefit in the intestine (and peripherally) by regulating the inflammatory function of various immune and non-immune cells (67). IL-8 is a potent neutrophil chemoattractant (68) and there is ample evidence that circulating IL-8 is nearly undetectable in healthy children (69) but elevated during infection (70–72). It was recently shown that IL-8 is abundantly produced by newborn but not adult *in vitro* stimulated T cells (73). The authors proposed that IL-8 performs a neonatal inflammatory function, compensating for low levels of other inflammatory cytokines in babies. Consistent with this previous study, we found T cells (and also B cells) from American children produced a strong IL-8 response upon stimulation (Supplementary Figure 3). In the context of the PROVIDE cohort, where enteropathogen exposure and intestinal alterations like environmental enteropathy are common, IL-8 and TGFβ likely play a critical role in gut health and wound healing. *In vitro* studies have shown that intestinal epithelial cells, in addition to making IL-8 in response to inflammatory stimuli, can also be stimulated by IL-8 through a CXCR-1 dependent mechanism (74). IL-8 and TGFβ both enhance migration of intestinal epithelial cells, a function required for efficient repair after physical damage to the barrier surface (74–76).

We hypothesize that the combination of enteric infection, subclinical environmental enteropathy, and the subsequent alterations to the intestinal barrier may explain the association (Figure 6B) observed between IL-8 and TGFβ production post-stimulation and stunting in Bangladeshi children. This hypothesis is consistent with two key pieces of evidence from prior work: (1) that markers of enhanced neutrophil recruitment and inflammation precede clinical malnutrition and stunting in the cohort (57), and (2) that malnourishment in Bangladeshi children is associated with an immature gut microbiome (77), possibly as a consequence of diarrhea as well as other factors (78). However, we note that our study was designed to identify associations with clinical stunting, not causes. Further work examining both IL-8 and TGFβ peripherally (from both PBMCs and serum cytokine analysis) as well as locally at the intestine along with the microbiome are needed to determine whether the stunting association observed reflects specific microbial alterations and host responses.

Another limitation of our study was that we were restricted to analyzing immune responses in peripheral blood. Oral vaccine failure is prevalent in low-income countries (7), while parenteral vaccines show no such defect. We were unable to identify any PBMC population that correlated with oral vaccine failure, suggesting that mucosa-specific responses are likely more important. Indeed, Naylor and colleagues determined that enteric rather than systemic inflammatory markers were better indicators of oral vaccine failure and stunting in the PROVIDE cohort (57). Since rates of environmental enteropathy are very different between high- and low-income countries, it would be of great interest to profile immune alterations at the gut interface. A point of future exploration is whether interventional strategies, such as improved sanitation and additional early immunization for enteroviruses, would have an effect on clinical factors like chronic infection and stunting, and whether there is a corresponding effect on pediatric immune development. One would expect that these strategies would reduce the rate of enteric infection, which might alter immune profiles to be more in line with those seen in more developed countries. Whether this would provide a benefit in terms of oral vaccine efficacy, immune, and overall health of children in countries like Bangladesh remains to be determined.

In summary, we present here a comparative study of early immune development in Bangladeshi vs. American children. While very similar at 1 year of age, marked differences are evident at 2 years of age and beyond, when Bangladeshi children show marked increases in effector T cells and other indicators of pathogen engagement. We also note a loss of IL-8 and TGFβ in healthier Bangladeshi 1-year-olds, perhaps associated with a reduced requirement for wound healing responses in the gut. Overall the results have implications for both the phenomenon of stunting, a major problem in poorer countries and are consistent with the Hygiene Hypothesis, with far more immune activity and accelerated immune maturation evident in Bangladeshi children than their American counterparts.

## Materials and Methods

### Study Enrollment and Sample Preparation

Samples were collected with ethics approval from all regulatory bodies involved. The PROVIDE study was performed in the Mirpur area of Dhaka, Bangladesh. A detailed explanation of the study design is published (34). Dhaka is densely populated with over a million inhabitants. Participants were selected from predominantly lower socioeconomic households living in slum conditions. A door-to-door community census was done to identify pregnant women; within seven days of birth, eligible infants were enrolled (eligibility criteria included no obvious congenital abnormalities or birth defects, no abnormal stools since birth, and no history of seizures or neurologic disorders) (34). Children were visited in their homes twice a week and a structured questionnaire administered that included diarrheal illness, fever, and use of antibiotics or oral rehydration solution. Nutritional status was measured at every study visit using a calibrated baby scale and supine length measurement equipment, with two complete measurements made at each time and the average reported. Subjects were tested for protozoa and rotavirus in stool samples when infants had diarrheal disease. For poliovirus and non-polio enterovirus surveillance, stool samples were tested on weeks 6, 14, and 52 of life. Children from the Bay Area in California, USA were enrolled at birth in the STORK study. English- or Spanish-speaking women with low-risk pregnancies and without non-gestational diabetes or other serious chronic illness were recruited prior to 36 weeks' gestation from several Bay Area clinics. Following the babies' births, mothers in STORK were interviewed weekly via automated phone call or email regarding disease symptoms in their babies. Infection surveillance was performed based on medical chart review. Whole blood samples from babies were obtained by venipuncture annually through age three (35). Adult control samples were collected from the Stanford Blood Bank (6 CMV negative, 1 CMV positive, and 1 CMV status unknown; mean age 50 years old; diverse ethnic backgrounds). Whole blood was collected into heparin or EDTA coated vacutainer tubes by standard venipuncture. For both studies, blood samples were maintained as close as possible to 21°C prior to processing and processing was performed within 4 h of draw. Peripheral blood mononuclear cells (PBMCs) were isolated by standard Ficoll density gradient centrifugation and cryopreserved until use. Samples from both study sites were maintained in long-term storage in liquid nitrogen and samples from Bangladesh were shipped in a nitrogen-charged dry shipper for transport to the United States to minimize study-specific storage and handling differences. Both studies enrolled participants on a rolling enrollment basis, from 2011 to 2014 for the PROVIDE study (with samples examined here collected from 2011 to 2016) and 2011 to 2015 for the STORK study (with samples from 2014 to 2015). In both cohorts, CMV seropositivity was determined by detecting CMV-specific IgG in serum or plasma by ELISA.

### CyTOF Antibody Reagents

Metal-conjugated antibodies were either purchased from Fluidigm or coupled in-house with DN3 or X8 polymers using the MaxPar antibody labeling kit (Fluidigm). For palladium coupling, metal was added to anti-CD45 antibodies with amine-reactive isothiocyanobenzyl-EDTA chelator, as described by Mei and colleagues (79). See Supplementary Table 2 for staining panel and clone details.

### Stimulation and Cell Staining

Cryopreserved PBMCs were thawed and washed in warmed, complete (R10) media (RPMI media with glutamax and HEPES (Gibco), 10% fetal bovine serum (Sigma), 1X penicillin/streptomycin, 1X non-essential amino acids, 1X sodium pyruvate). Cells were rested for 1 h at 37°C in R10 with benzonase (Sigma), then enumerated and assessed for viability by trypan blue exclusion; any samples with <70% live cells after the 1 h resting period were excluded from data analysis. Samples were split in half (up to five million live PBMCs) for use in stimulation assays. Five million live PBMCs (or half of the total if <10 million cells were available) were washed and resuspended either in 500 μl of R10 (for non-stimulated controls), or R10 with 1X cell stimulation cocktail (containing PMA-ionomycin, eBioscience) and maintained in 24-well plates (BD Biosciences). Anti-CD107a and anti-CD107b metal-coupled antibodies were added to all samples at a final concentration of 0.5 μg/ml each. PBMCs were incubated for 1 h at 37°C. Then, 1X protein transport inhibitor cocktail (containing monensin and brefeldin, eBioscience) was added to all samples. PBMCs were further incubated for 5 h at 37°C.

After a total of 6 h of stimulation, PBMCs were harvested for staining. Cells were washed two times with CyFACS buffer (PBS with 0.1% w/v bovine serum albumin, 2 mM EDTA, and 0.05% v/v sodium azide), then barcoded with anti-CD45 antibodies coupled to Pd104, Pd106, Pd108, and In113 as previously described (79), using combinations of two or three metals to define one barcode. Barcode staining was completed on ice for 30 min, then samples were washed three times with CyFACS buffer and compatible barcoded samples were pooled (up to 10 samples per pool). Each barcoded pool included a control PBMC sample from a local adult blood bank donor to control for potential differences in staining between pools. Samples were surface stained with primary antibodies for 30 min on ice. After further washing, dead cells were detected with cisplatin (Fluidigm) diluted 1/1,000 in PBS for 5 min at room temperature. Samples were washed two times with CyFACS buffer, then fixed overnight at 4°C with 2% paraformaldehyde (Electron Microscopy Sciences) diluted in PBS. The following day, samples were washed and permeabilized with 1X permeabilization buffer (eBioscience), then stained with intracellular antibodies for 30 min on ice. After further washes, samples were resuspended in 1X iridium DNA intercalator (Fluidigm) diluted in PBS containing 2% paraformaldehyde. Intercalator staining was done on ice for 30 min. Finally, PBMCs were washed once with perm buffer, twice with PBS, and three times with water. Prior to acquisition, samples were diluted to ~7.5 × 10^5^ cells/ml in water with 10% v/v four-element calibration bead solution (Fluidigm). Data were collected on a CyTOF2 instrument (Fluidigm). Signal normalization using calibration beads was done with the built-in CyTOF2 software.

### Manual Gating

Manual gating was done using Flowjo (TreeStar) software. First, normalized fcs files were gated on event length, DNA intercalator staining to discriminate singlets from doublets, then on live cells. Barcodes were deconvoluted and individual samples were exported as separate fcs files for manual gating and automated clustering analysis. Samples with <3,000 live intact singlet cells were discarded from the analysis. See Supplementary Figure 2 for sample gating strategy.

### Dimensionality Reduction and Automated Gating

In order to accommodate the large size of the dataset, computational down-sampling was used to draw 6,000 live cells from each data file (donor, time point, and stimulation), as well as an additional 30,000 cells from the healthy adult controls. We opted to include some cells from healthy adults so that we could identify cell types that were infrequent in both PROVIDE and STORK cohorts, presumably as a consequence of age. In total, about 375,000 cell events were included for each clustering analysis. A subset of phenotypic markers (*n* = 23 markers for unstimulated; *n* = 32 markers for stimulated) was selected from the total CyTOF panel (Supplementary Table 2), and dimensionality reduction was performed using t-Distributed Stochastic Neighbor Embedding (t-SNE) (80, 81), with a perplexity of 60, theta of 0.5, and 2,000 iterations.

After dimensionality reduction, a novel clustering algorithm (referred to as AdjClust) was developed to define individual subsets of cells. Briefly, AdjClust uses hierarchical clustering of the original n-dimensional cell data, with the added stipulation that the cells must be “adjacent” in 2-dimensional space. The 2-dimensional t-SNE map was first partitioned into equal-sized hexagonal bins (~10,000 in total), and the marker values of all cells within a bin were averaged to get a “bin expression” value. Bins that did not contain any cells were dropped from the analysis. Pairwise distances between two bins, p and q, were calculated using the following formula:

$$\begin{array}{l} d\left(p,q\right)=\sqrt{\overset{n}{\underset{i\;=\;1}{\sum }}w_{i}{\left(p_{i}-q_{i}\right)}^{2}} \end{array}$$

where w_i_ is a manually-defined weighting value for marker *i*. A complete list of manually-assigned weights for the markers in the AdjClust analyses can be found in Supplementary Table 3.

Using the resulting distance matrix, complete-linkage hierarchical clustering was performed, with the added stipulation that, at each combination step, two clusters can only be combined if at least one hexagonal bin within each cluster is directly “adjacent” to a bin in the other cluster. Hexagons were defined as adjacent if either: (A) they shared a border with each other, or (B) if a bin had no non-empty neighbors, then the closest bins were defined as neighbors. Adjacency clustering was performed until no “adjacent” clusters remained; at which point the final clustering steps were completed using standard complete-linkage hierarchical clustering. The resulting tree was cut to the desired number of clusters, and then cells within each hexagonal bin were assigned to the same cluster as the bin. A minimum cluster size of 1,500 cells was manually assigned.

### Statistical Analysis

All statistical analyses except the multivariable clinical correlation analysis were done using R (82). To evaluate diarrheal disease burden between the PROVIDE and STORK cohorts, a linear mixed model was used to correct for multiple measurements from repeat subjects. For analyses where cell clusters or manually gated populations were compared between Bangladeshi and American children of similar ages, Mann-Whitney non-parametric tests were used and the resulting *p*-values were adjusted for multiple hypothesis testing using the Benjamini and Hochberg (BH) false discovery rate method, with a cutoff of FDR < 0.1 used for consideration.

For evaluating variance associated with clinical features, PCA was applied to the automated clustering cell count datasets for dimensionality reduction and all but the final dimension were retained. The principal components were then modeled as a function of demographic and other variables (batch number, age, location, ethnic background, sex, CMV status) using a linear multivariate multiple regression model. Partial percent variance explained (partial eta^2^) was calculated using the “etasq” function in the “heplots” package (version 1.3-4), which calculates the percent variance explained for each variable from the Pillai statistic. A type-II MANOVA test was performed on the linear model using the “Anova” function from the “car” R package (version 2.1-5), and *p*-values for the contribution of each variable were calculated from the Pillai test statistic.

To explore the potential relationship between immune cell functions and stunting, we performed several filtering steps followed by a permutation analysis. First, outliers were removed based on k-means clustering on the CyTOF data acquired from stimulated and unstimulated samples. Only samples that clustered with the appropriate stimulation condition were included in the analysis to eliminate possible complications from donors actively responding to an acute infection. Next, we divided the qualifying samples into three groups: five samples from American children (group 0; HAZ values were estimated to be 0), eight samples from non-stunted (HAZ > −1.5) Bangladeshi children (group 1), and eight samples from more stunted (HAZ < = −1.5) Bangladeshi children (group 2). We then considered the following event:

$$\begin{array}{l} \frac{\mu_{i0}-\mu_{i1}}{s_{i,0}}\geq \theta ,\frac{\mu_{i2}-\mu_{i1}}{s_{i,2}}\geq \theta \end{array}$$

where μ_*ij*_ is the group mean for group *j* and feature *j*, and *s*_*i, j*_ is the pooled standard deviation for group *j* and group 1. The cell populations were filtered based on whether they satisfy the above criterion. For different cut-offs θ, we estimate the *p*-value and FDR for selected features based on permutation testing. To account for cytokine and cell population correlations within a single individual, HAZ scores were permuted 5,000 times and a cutoff of *p* = 0.05 and FDR = 0.2 was established. Since both IL-8 and TGFβ were identified across multiple cell populations with this cutoff, we first validated these two markers on the training data. Using the first component from a principal component analysis to perform a linear regression with one-sided *t*-tests, we calculated *p*-values for IL-8 (*p* = 0.24; first principal component explains 75% of total variation) and TGFβ (*p* = 0.04; first principal component explains 86% of total variation). Nine independent samples from the PROVIDE study were run to serve as a validation set for IL-8 and TGFβ; we observe *p* = 0.037 and *p* = 0.043, respectively, with a one-sided test. The combined *p*-values from both training and validation data are *p* = 0.009 (IL-8) and *p* = 0.002 (TGFβ).

## Data Availability Statement

The datasets generated and analyzed in this study can be found in the Flow Repository database (Experiment ID: FR-FCM-ZYV8). The Adjacency Clustering (AdjClust) algorithm can be downloaded from GitHub at https://bitbucket.org/cbolen1/adjclust/.

## Ethics Statement

This study was carried out in accordance with the recommendations of the World Health Organization and National Institutes of Health guidelines by Research Review Committee and Ethics Review Committee at the ICDDRB,B and at Institutional Review Boards at the University of Virginia, University of Vermont, and Stanford University with written informed consent from all literate subjects. The consent process for illiterate subjects was approved by the same review committees and is described in detail in Kirkpatrick et al. (34). All literate subjects gave written informed consent in accordance with the Declaration of Helsinki. The protocol was approved by the Research Review Committee and Ethics Review Committee at the ICDDRB,B and at Institutional Review Boards at the University of Virginia, University of Vermont, and Stanford University.

## Author Contributions

LW, NS, and CL designed and performed experiments, collected, and analyzed data. CB designed the clustering algorithm and analyzed data. LW and CB did statistical analysis. LG and RT performed the stunting clinical correlation analysis. BK, RH, JP, and WP led study design, sample and clinical data collection and logistics, and oversaw clinical assays. LW, WP, and MD conceived of the study and guided it throughout. LW, CB, CL, JP, WP, and MD wrote the manuscript.

### Conflict of Interest

The authors declare that the research was conducted in the absence of any commercial or financial relationships that could be construed as a potential conflict of interest.

## References

[1] Basha S Surendran N Pichichero M. Immune responses in neonates. Expert Rev Clin Immunol. (2014) 10:1171–84. 10.1586/1744666X.2014.942288 25088080 PMC4407563

[2] MacGillivray DM Kollmann TR. The role of environmental factors in modulating immune responses in early life. Front Immunol. (2014) 5:434. 10.3389/fimmu.2014.00434 25309535 PMC4161944

[3] Simon AK Hollander GA McMichael A. Evolution of the immune system in humans from infancy to old age. Proc Biol Sci. (2015) 282:20143085. 10.1098/rspb.2014.3085 26702035 PMC4707740

[4] Casanova JL Abel L. Inborn errors of immunity to infection: the rule rather than the exception. J Exp Med. (2005) 202:197–201. 10.1084/jem.20050854 16027233 PMC2212996

[5] Su LF Davis MM. Antiviral memory phenotype T cells in unexposed adults. Immunol Rev. (2013) 255:95–109. 10.1111/imr.12095 23947350

[6] Veitch AM Kelly P Zulu IS Segal I Farthing MJ. Tropical enteropathy: a T-cell-mediated crypt hyperplastic enteropathy. Eur J Gastroenterol Hepatol. (2001) 13:1175–81. 10.1097/00042737-200110000-00009 11711773

[7] Korpe PS Petri WA Jr. Environmental enteropathy: critical implications of a poorly understood condition. Trends Mol Med. (2012) 18:328–36. 10.1016/j.molmed.2012.04.007 22633998 PMC3372657

[8] Guerrant RL DeBoer MD Moore SR Scharf RJ Lima AA The impoverished gut–a triple burden of diarrhoea, stunting and chronic disease. Nat Rev Gastroenterol Hepatol. (2013) 10:220–9. 10.1038/nrgastro.2012.239 23229327 PMC3617052

[9] Lindenbaum J Kent TH Sprinz H. Malabsorption and jejunitis in American Peace Corps volunteers in Pakistan. Ann Intern Med. (1966) 65:1201–9. 10.7326/0003-4819-65-6-1201 5928480

[10] Brown EM Wlodarska M Willing BP Vonaesch P Han J Reynolds LA. Diet and specific microbial exposure trigger features of environmental enteropathy in a novel murine model. Nat Commun. (2015) 6:7806. 10.1038/ncomms8806 26241678 PMC4532793

[11] Campbell DI Murch SH Elia M Sullivan PB Sanyang MS Jobarteh B. Chronic T cell-mediated enteropathy in rural west African children: relationship with nutritional status and small bowel function. Pediatr Res. (2003) 54:306–11. 10.1203/01.PDR.0000076666.16021.5E 12788978

[12] Mondal D Minak J Alam M Liu Y Dai J Korpe P. Contribution of enteric infection, altered intestinal barrier function, and maternal malnutrition to infant malnutrition in Bangladesh. Clin Infect Dis. (2012) 54:185–92. 10.1093/cid/cir807 22109945 PMC3245731

[13] Hayward AR. The human fetus and newborn: development of the immune response. Birth Defects Orig Artic Ser. (1983) 19:289–94. 6606446

[14] Levy O. Innate immunity of the newborn: basic mechanisms and clinical correlates. Nat Rev Immunol. (2007) 7:379–90. 10.1038/nri2075 17457344

[15] Ygberg S Nilsson A. The developing immune system - from foetus to toddler. Acta Paediatr. (2012) 101:120–7. 10.1111/j.1651-2227.2011.02494.x 22003882

[16] Rook GA Dheda K Zumla A. Immune systems in developed and developing countries; implications for the design of vaccines that will work where BCG does not. Tuberculosis. (2006) 86:152–62. 10.1016/j.tube.2006.01.018 16510309

[17] Vesikari T Matson DO Dennehy P Van Damme P Santosham M Rodriguez Z. Safety and efficacy of a pentavalent human-bovine (WC3) reassortant rotavirus vaccine. N Engl J Med. (2006) 354:23–33. 10.1056/NEJMoa052664 16394299

[18] Patel M Pedreira C De Oliveira LH Tate J Orozco M Mercado J. Association between pentavalent rotavirus vaccine and severe rotavirus diarrhea among children in Nicaragua. JAMA. (2009) 301:2243–51. 10.1001/jama.2009.756 19491186

[19] Madhi SA Cunliffe NA Steele D Witte D Kirsten M Louw C. Effect of human rotavirus vaccine on severe diarrhea in African infants. N Engl J Med. (2010) 362:289–98. 10.1056/NEJMoa0904797 20107214

[20] Barreiro LB Quintana-Murci L. From evolutionary genetics to human immunology: how selection shapes host defence genes. Nat Rev Genet. (2010) 11:17–30. 10.1038/nrg2698 19953080

[21] Nguyen M Leuridan E Zhang T De Wit D Willems F Van Damme P. Acquisition of adult-like TLR4 and TLR9 responses during the first year of life. PLoS ONE. (2010) 5:e10407. 10.1371/journal.pone.0010407 20442853 PMC2861003

[22] Burl S Townend J Njie-Jobe J Cox M Adetifa UJ Touray E. Age-dependent maturation of Toll-like receptor-mediated cytokine responses in Gambian infants. PLoS ONE. (2011) 6:e18185. 10.1371/journal.pone.0018185 21533209 PMC3076452

[23] Teran R Mitre E Vaca M Erazo S Oviedo G Hubner MP. Immune system development during early childhood in tropical Latin America: evidence for the age-dependent down regulation of the innate immune response. Clin Immunol. (2011) 138:299–310. 10.1016/j.clim.2010.12.011 21247809 PMC3043252

[24] Lisciandro JG Prescott SL Nadal-Sims MG Devitt CJ Pomat W Siba PM. Ontogeny of Toll-like and NOD-like receptor-mediated innate immune responses in Papua New Guinean infants. PLoS ONE. (2012) 7:e36793. 10.1371/journal.pone.0036793 22649499 PMC3359332

[25] Reikie BA Adams RC Ruck CE Ho K Leligdowicz A Pillay S. Ontogeny of Toll-like receptor mediated cytokine responses of South African infants throughout the first year of life. PLoS ONE. (2012) 7:e44763. 10.1371/journal.pone.0044763 23028609 PMC3441420

[26] Kollmann TR. Variation between populations in the innate immune response to vaccine adjuvants. Front Immunol. (2013) 4:81. 10.3389/fimmu.2013.00081 23565115 PMC3613898

[27] Nedelec Y Sanz J Baharian G Szpiech ZA Pacis A Dumain A. Genetic ancestry and natural selection drive population differences in immune responses to pathogens. Cell. (2016) 167:657–69 e621. 10.1016/j.cell.2016.09.025 27768889

[28] Corbett NP Blimkie D Ho KC Cai B Sutherland DP Kallos A. Ontogeny of Toll-like receptor mediated cytokine responses of human blood mononuclear cells. PLoS ONE. (2010) 5:e15041. 10.1371/journal.pone.0015041 21152080 PMC2994830

[29] Djuardi Y Wammes LJ Supali T Sartono E Yazdanbakhsh M. Immunological footprint: the development of a child's immune system in environments rich in microorganisms and parasites. Parasitology. (2011) 138:1508–18. 10.1017/S0031182011000588 21767432

[30] Cooper PJ Amorim LD Figueiredo CA Esquivel R Tupiza F Erazo S. Effects of environment on human cytokine responses during childhood in the tropics: role of urban versus rural residence. World Allergy Organ J. (2015) 8:22. 10.1186/s40413-015-0071-2 26312126 PMC4527255

[31] Hussey GD Watkins ML Goddard EA Gottschalk S Hughes EJ Iloni K. Neonatal mycobacterial specific cytotoxic T-lymphocyte and cytokine profiles in response to distinct BCG vaccination strategies. Immunology. (2002) 105:314–24. 10.1046/j.1365-2567.2002.01366.x 11918693 PMC1782661

[32] Rook GA Dheda K Zumla A. Immune responses to tuberculosis in developing countries: implications for new vaccines. Nat Rev Immunol. (2005) 5:661–7. 10.1038/nri1666 16056257

[33] Strachan DP. Hay fever, hygiene, and household size. BMJ. (1989) 299:1259–60. 10.1136/bmj.299.6710.1259 2513902 PMC1838109

[34] Kirkpatrick BD Colgate ER Mychaleckyj JC Haque R Dickson DM Carmolli MP. The performance of rotavirus and oral Polio Vaccines in Developing Countries (PROVIDE) study: description of methods of an interventional study designed to explore complex biologic problems. Am J Trop Med Hyg. (2015) 92:744–51. 10.4269/ajtmh.14-0518 25711607 PMC4385767

[35] Ley C Sanchez Mde L Mathur A Yang S Sundaram V Parsonnet J. Stanford's Outcomes Research in Kids (STORK): a prospective study of healthy pregnant women and their babies in Northern California. BMJ Open. (2016) 6:e010810. 10.1136/bmjopen-2015-010810 27075843 PMC4838723

[36] Pindyck T Tate JE Parashar UD. A decade of experience with rotavirus vaccination in the United States - vaccine uptake, effectiveness, and impact. Expert Rev Vaccines. (2018) 17:593–606. 10.1080/14760584.2018.1489724 29909693 PMC9199965

[37] Beer KD Gargano JW Roberts VA Hill VR Garrison LE Kutty PK. Surveillance for waterborne disease outbreaks associated with drinking water - United States, 2011-2012. Morb Mortal Wkly Rep. (2015) 64:842–8. 10.15585/mmwr.mm6431a2 26270059 PMC4584589

[38] McClung RP Roth DM Vigar M Roberts VA Kahler AM Cooley LA. Waterborne disease outbreaks associated with environmental and undetermined exposures to water - United States, 2013-2014. Morb Mortal Wkly Rep. (2017) 66:1222–5. 10.15585/mmwr.mm6644a4 29120997 PMC5679586

[39] Haque R Mondal D Duggal P Kabir M Roy S Farr BM. Entamoeba histolytica infection in children and protection from subsequent amebiasis. Infect Immun. (2006) 74:904–9. 10.1128/IAI.74.2.904-909.2006 16428733 PMC1360358

[40] Kotloff KL Nataro JP Blackwelder WC Nasrin D Farag TH Panchalingam S. Burden and aetiology of diarrhoeal disease in infants and young children in developing countries (the Global Enteric Multicenter Study, GEMS): a prospective, case-control study. Lancet. (2013) 382:209–22. 10.1016/S0140-6736(13)60844-2 23680352

[41] Taniuchi M Sobuz SU Begum S Platts-Mills JA Liu J Yang Z. Etiology of diarrhea in Bangladeshi infants in the first year of life analyzed using molecular methods. J Infect Dis. (2013) 208:1794–802. 10.1093/infdis/jit507 24041797 PMC3814844

[42] Gilchrist CA Petri SE Schneider BN Reichman DJ Jiang N Begum S. Role of the gut microbiota of children in diarrhea due to the protozoan parasite *Entamoeba histolytica*. J Infect Dis. (2015) 213:1579–85. 10.1093/infdis/jiv772 26712950 PMC4837909

[43] Amir el AD Davis KL Tadmor MD Simonds EF Levine JH Bendall SC viSNE enables visualization of high dimensional single-cell data and reveals phenotypic heterogeneity of leukemia. Nat Biotechnol. (2013) 31:545–52. 10.1038/nbt.2594 23685480 PMC4076922

[44] Becher B Schlitzer A Chen J Mair F Sumatoh HR Teng KW. High-dimensional analysis of the murine myeloid cell system. Nat Immunol. (2014) 15:1181–9. 10.1038/ni.3006 25306126

[45] Shekhar K Brodin P Davis MM Chakraborty AK. Automatic Classification of Cellular Expression by Nonlinear Stochastic Embedding (ACCENSE). Proc Natl Acad Sci USA. (2014) 111:202–7. 10.1073/pnas.1321405111 24344260 PMC3890841

[46] Chester C Maecker HT. Algorithmic Tools for Mining High-Dimensional Cytometry Data. J Immunol. (2015) 195:773–9. 10.4049/jimmunol.1500633 26188071 PMC4507289

[47] Brenchley JM Karandikar NJ Betts MR Ambrozak DR Hill BJ Crotty LE. Expression of CD57 defines replicative senescence and antigen-induced apoptotic death of CD8+ T cells. Blood. (2003) 101:2711–20. 10.1182/blood-2002-07-2103 12433688

[48] Brodin P Jojic V Gao T Bhattacharya S Angel CJ Furman D. Variation in the human immune system is largely driven by non-heritable influences. Cell. (2015) 160:37–47. 10.1016/j.cell.2014.12.020 25594173 PMC4302727

[49] Klenerman P Oxenius A. T cell responses to cytomegalovirus. Nat Rev Immunol. (2016) 16:367–77. 10.1038/nri.2016.38 27108521

[50] Barreiro LB Laval G Quach H Patin E Quintana-Murci L. Natural selection has driven population differentiation in modern humans. Nat Genet. (2008) 40:340–5. 10.1038/ng.78 18246066

[51] Vasseur E Boniotto M Patin E Laval G Quach H Manry J. The evolutionary landscape of cytosolic microbial sensors in humans. Am J Hum Genet. (2012) 91:27–37. 10.1016/j.ajhg.2012.05.008 22748209 PMC3397270

[52] Quach H Rotival M Pothlichet J Loh YE Dannemann M Zidane N. Genetic adaptation and neandertal admixture shaped the immune system of human populations. Cell. (2016) 167:643–56 e617. 10.1016/j.cell.2016.09.024 27768888 PMC5075285

[53] Barreiro LB Ben-Ali M Quach H Laval G Patin E Pickrell JK. Evolutionary dynamics of human Toll-like receptors and their different contributions to host defense. PLoS Genet. (2009) 5:e1000562. 10.1371/journal.pgen.1000562 19609346 PMC2702086

[54] Wlasiuk G Khan S Switzer WM Nachman MW. A history of recurrent positive selection at the toll-like receptor 5 in primates. Mol Biol Evol. (2009) 26:937–49. 10.1093/molbev/msp018 19179655 PMC2734149

[55] Fumagalli M Cagliani R Riva S Pozzoli U Biasin M Piacentini L. Population genetics of IFIH1: ancient population structure, local selection, and implications for susceptibility to type 1 diabetes. Mol Biol Evol. (2010) 27:2555–66. 10.1093/molbev/msq141 20538742

[56] Quintana-Murci L Clark AG. Population genetic tools for dissecting innate immunity in humans. Nat Rev Immunol. (2013) 13:280–93. 10.1038/nri3421 23470320 PMC4015519

[57] Naylor C Lu M Haque R Mondal D Buonomo E Nayak U. Environmental enteropathy, oral vaccine failure and growth faltering in infants in Bangladesh. EBio Med. (2015) 2:1759–66. 10.1016/j.ebiom.2015.09.036 26870801 PMC4740306

[58] Tu W Chen S Sharp M Dekker C Manganello AM Tongson EC. Persistent and selective deficiency of CD4+ T cell immunity to cytomegalovirus in immunocompetent young children. J Immunol. (2004) 172:3260–7. 10.4049/jimmunol.172.5.3260 14978134

[59] Appay V Dunbar PR Callan M Klenerman P Gillespie GM Papagno L. Memory CD8+ T cells vary in differentiation phenotype in different persistent virus infections. Nat Med. (2002) 8:379–85. 10.1038/nm0402-379 11927944

[60] Pitcher CJ Hagen SI Walker JM Lum R Mitchell BL Maino VC. Development and homeostasis of T cell memory in rhesus macaque. J Immunol. (2002) 168:29–43. 10.4049/jimmunol.168.1.29 11751943

[61] Sylwester AW Mitchell BL Edgar JB Taormina C Pelte C Ruchti F. Broadly targeted human cytomegalovirus-specific CD4+ and CD8+ T cells dominate the memory compartments of exposed subjects. J Exp Med. (2005) 202:673–85. 10.1084/jem.20050882 16147978 PMC2212883

[62] Furman D Jojic V Sharma S Shen-Orr SS Angel CJ Onengut-Gumuscu S. Cytomegalovirus infection enhances the immune response to influenza. Sci Transl Med. (2015) 7:281ra243. 10.1126/scitranslmed.aaa2293 25834109 PMC4505610

[63] Bhutta ZA Ahmed T Black RE Cousens S Dewey K Giugliani E. What works? Interventions for maternal and child undernutrition and survival. Lancet. (2008) 371:417–40. 10.1016/S0140-6736(07)61693-6 18206226

[64] Najera O Gonzalez C Toledo G Lopez L Ortiz R. Flow cytometry study of lymphocyte subsets in malnourished and well-nourished children with bacterial infections. Clin Diagn Lab Immunol. (2004) 11:577–80. 10.1128/CDLI.11.3.577-580.2004 15138185 PMC404584

[65] Gorelik L Flavell RA. Transforming growth factor-beta in T-cell biology. Nat Rev Immunol. (2002) 2:46–53. 10.1038/nri704 11905837

[66] Li MO Wan YY Sanjabi S Robertson AK Flavell RA. Transforming growth factor-beta regulation of immune responses. Annu Rev Immunol. (2006) 24:99–146. 10.1146/annurev.immunol.24.021605.090737 16551245

[67] Smith PD Smythies LE Shen R Greenwell-Wild T Gliozzi M Wahl SM. Intestinal macrophages and response to microbial encroachment. Mucosal Immunol. (2011) 4:31–42. 10.1038/mi.2010.66 20962772 PMC3821935

[68] Huber AR Kunkel SL Todd RF III Weiss SJ. Regulation of transendothelial neutrophil migration by endogenous interleukin-8. Science. (1991) 254:99–102. 10.1126/science.1718038 1718038

[69] Sack U Burkhardt U Borte M Schadlich H Berg K Emmrich F. Age-dependent levels of select immunological mediators in sera of healthy children. Clin Diagn Lab Immunol. (1998) 5:28–32. 9455875 10.1128/cdli.5.1.28-32.1998 PMC121386

[70] Franz AR Steinbach G Kron M Pohlandt F. Interleukin-8: a valuable tool to restrict antibiotic therapy in newborn infants. Acta Paediatr. (2001) 90:1025–32. 10.1111/j.1651-2227.2001.tb01359.x 11683191

[71] Mehr SS Doyle LW Rice GE Vervaart P Henschke P. Interleukin-6 and interleukin-8 in newborn bacterial infection. Am J Perinatol. (2001) 18:313–24. 10.1055/s-2001-17857 11607849

[72] Simonsen KA Anderson-Berry AL Delair SF Davies HD. Early-onset neonatal sepsis. Clin Microbiol Rev. (2014) 27:21–47. 10.1128/CMR.00031-13 24396135 PMC3910904

[73] Gibbons D Fleming P Virasami A Michel ML Sebire NJ Costeloe K. Interleukin-8 (CXCL8) production is a signatory T cell effector function of human newborn infants. Nat Med. (2014) 20:1206–10. 10.1038/nm.3670 25242415

[74] Sturm A Baumgart DC d'Heureuse JH Hotz A Wiedenmann B Dignass AU. CXCL8 modulates human intestinal epithelial cells through a CXCR1 dependent pathway. Cytokine. (2005) 29:42–8. 10.1016/j.cyto.2004.09.007 15579377

[75] Dignass AU Podolsky DK. Cytokine modulation of intestinal epithelial cell restitution: central role of transforming growth factor beta. Gastroenterology. (1993) 105:1323–32. 10.1016/0016-5085(93)90136-Z 8224636

[76] Dignass AU. Mechanisms and modulation of intestinal epithelial repair. Inflamm Bowel Dis. (2001) 7:68–77. 10.1097/00054725-200102000-00014 11233665

[77] Subramanian S Huq S Yatsunenko T Haque R Mahfuz M Alam MA. Persistent gut microbiota immaturity in malnourished Bangladeshi children. Nature. (2014) 510:417–21. 10.1038/nature13421 24896187 PMC4189846

[78] David LA Weil A Ryan ET Calderwood SB Harris JB Chowdhury F. Gut microbial succession follows acute secretory diarrhea in humans. MBio. (2015) 6:e00381–00315. 10.1128/mBio.00381-15 25991682 PMC4442136

[79] Mei HE Leipold MD Schulz AR Chester C Maecker HT. Barcoding of live human peripheral blood mononuclear cells for multiplexed mass cytometry. J Immunol. (2015) 194:2022–31. 10.4049/jimmunol.1402661 25609839 PMC4323739

[80] van der Maaten LHG Visualizing Data using t-SNE. J Mach Learn Res. (2008) 9:2579–605.

[81] van der Maaten L Accelerating t-SNE using Tree-Based Algorithms. J Mach Learn Res. (2014) 15:3221–45.

[82] R Core Team (2015). R: A Language and Environment for Statistical Computing. R Foundation for Statistical Computing. Available online at: http://www.R-project.org/ (accessed December 1, 2018).

