# Supplementary material for: Increased T Cell Differentiation and Cytolytic Function in Bangladeshi Compared to American Children
Source: Front Immunol. 2019 Sep 20;10:2239. doi: 10.3389/fimmu.2019.02239 (PMC6763580; doi:10.3389/fimmu.2019.02239)
Supplement: Supplementary file 1 [file Data_Sheet_1.pdf]

Supplementary Material

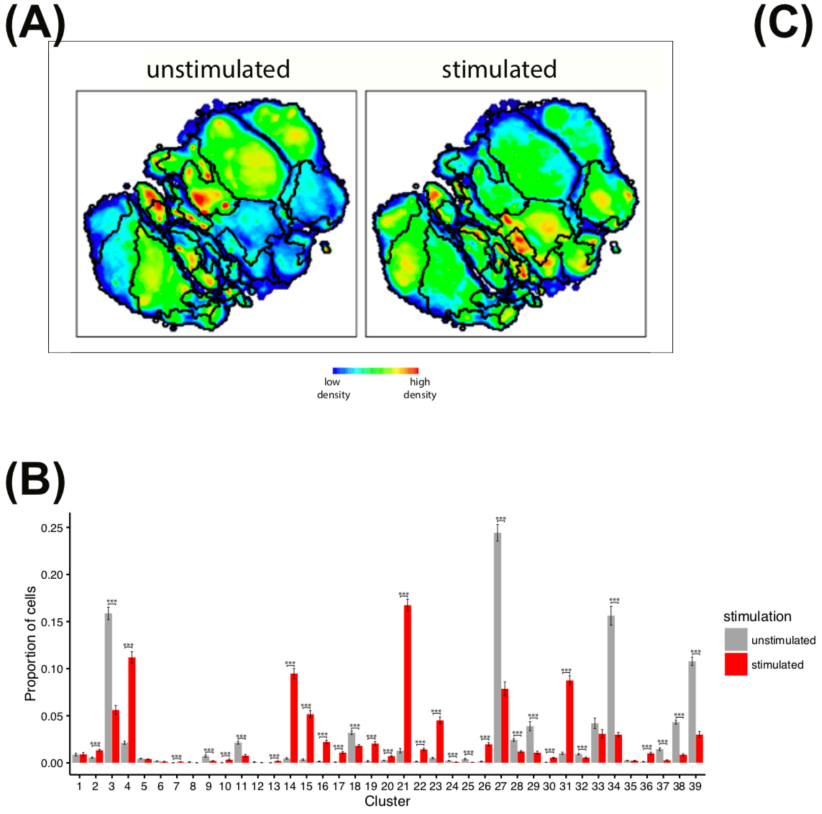

(C)

Cluster	Identity
1	memory B cells
2	CD69+ memory B cells
3	naïve B cells
4	CD69+ naïve B cells
5	CD69+ IgM B cells
6	CD25+ uncharacterized
7	CD69+ CD25+ IL-2+ uncharacterized
8	mixed
9	undefined APCs
10	CD57+ mixed cytokines CD4 T cells
11	CD56+ CD57+ NK cells
12	undefined APCs
13	IL-8+ γδ T cells
14	IL-2+ TGFβ+ mixed TNF+ IFNγ+ CD4 T cells
15	IL-8+ naïve CD4 T cells
16	CD69+ mixed cytokines naïve CD8 T cells
17	CD69+ mixed cytokines naïve CD8 T cells
18	CD56+ CD57- NK cells
19	IFNγ+ MIP-1β+ CD57+ memory CD8 T cells
20	IFNγ+ MIP-1β+ CD57+ memory CD8 T cells
21	CD69+ CD40L+ CD4 T cells
22	IL-2+ IL-8+ naïve CD4 T cells
23	IL-8+ naïve B cells
24	naïve B cells
25	CD57+ memory CD4 T cells
26	CD56+ mixed cytokines NK cells
27	naïve CD4 T cells
28	CD27+ CD57- γδ T cells
29	monocytes
30	IL-2+ naïve CD8 T cells
31	CD69+ naïve CD8 T cells
32	perforin+ CD57+ γδ T cells
33	MIP-1β+ IL-8+ monocytes
34	mixed Treg TFH memory CD4 T cells
35	CCR7- CD8 T cells
36	CD69+ mixed cytokines gd T cells
37	CD57+ perforin+ EMRA CD8 T cells
38	CD27+ memory CD8 T cells
39	naïve CD8 T cells

Supplementary Figure 1. Adjacency Clustering effectively separates stimulated and unstimulated PBMC samples. (A) AdjClust maps generated from unstimulated (left) and PMA-ionomcyin stimulated (right) PBMC samples. CyTOF data includes samples from Bangladeshi children, American children, and American adults. **(B)** Frequency of live cells populating each cluster in unstimulated (grey) and stimulated (red) conditions. Significance levels were determined by Student's unpaired t tests followed by BH adjustment for multiple testing. All significant FDR values (***) were less than 0.001. Values plotted are means +/- SEM. **(C)** Cluster profiles as determined by manual assignment.

Pooled, barcoded FCS files were pre-gated on event length, DNA intercalator, live cells, then debarcoded and exported as individual FCS files. Main population markers:

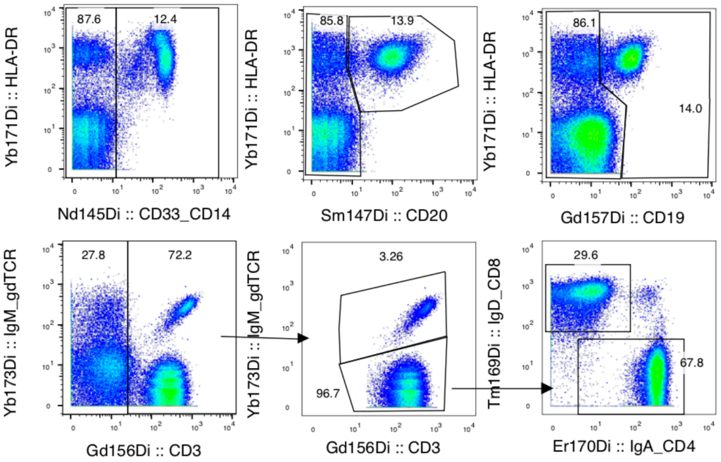

main population gates (Boolean)

Monocytes: CD33+|CD14+, CD20-CD19-CD56-CD3-
B cells: CD19+CD20+/dimHLA-DR+, CD33-CD14-CD3-CD56-CD16-
NK cells: CD56+|CD16+, CD33-CD14-CD20-CD19-CD3-
T cells: CD3+, CD33-CD14-CD20-CD19-CD16-
Gamma delta T cells: gdTCR+
CD4 T cells: gdTCR- CD4+
CD8 T cells: gdTCR- CD8+

sample CD4 T cell gating (unstimulated)

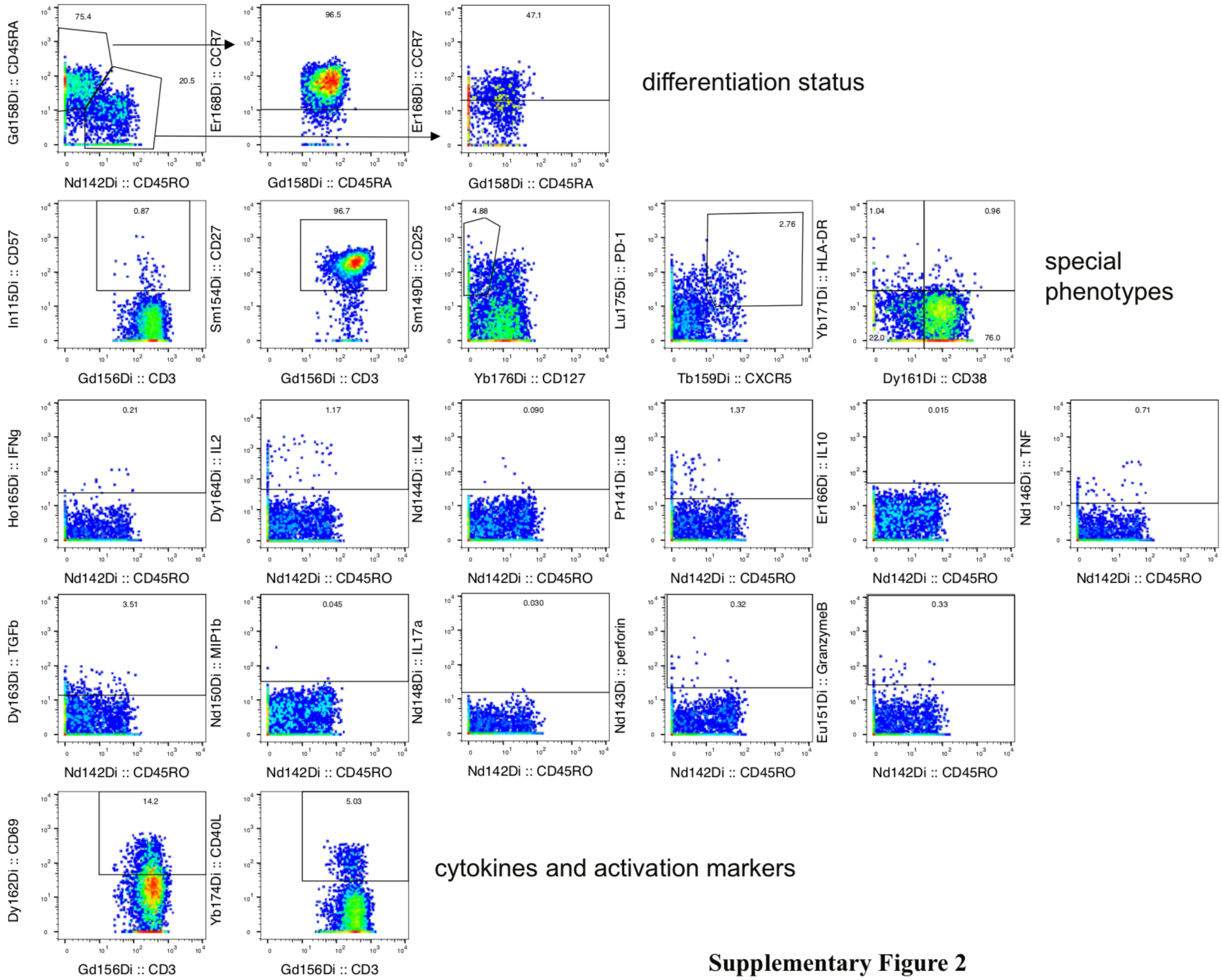

Supplementary Figure 2
see next page for stimulated example and legend

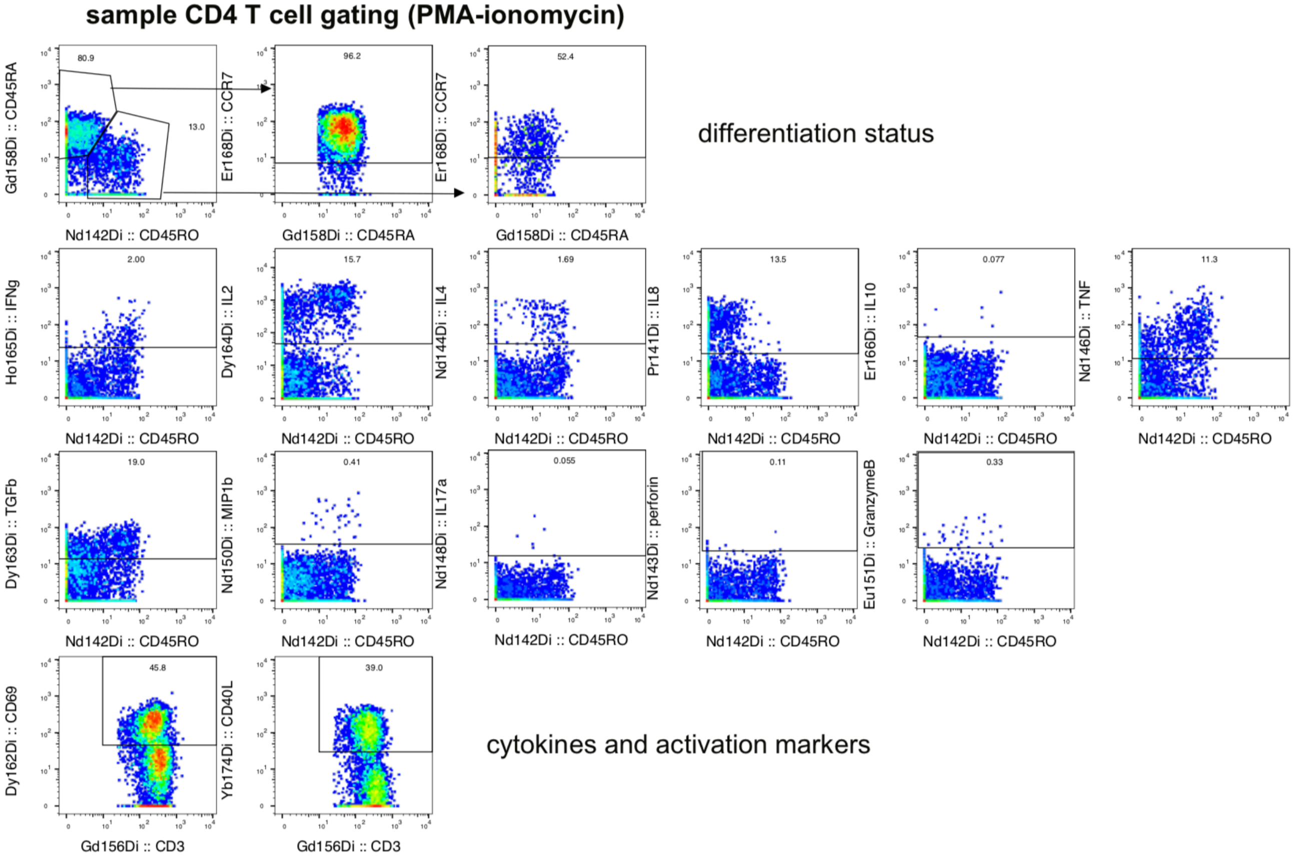

Supplementary Figure 2. Manual gating strategy for main cell populations with Boolean gating strategy and sample staining from CD4 T cells for phenotype and cytokine analysis in unstimulated and stimulated cells.

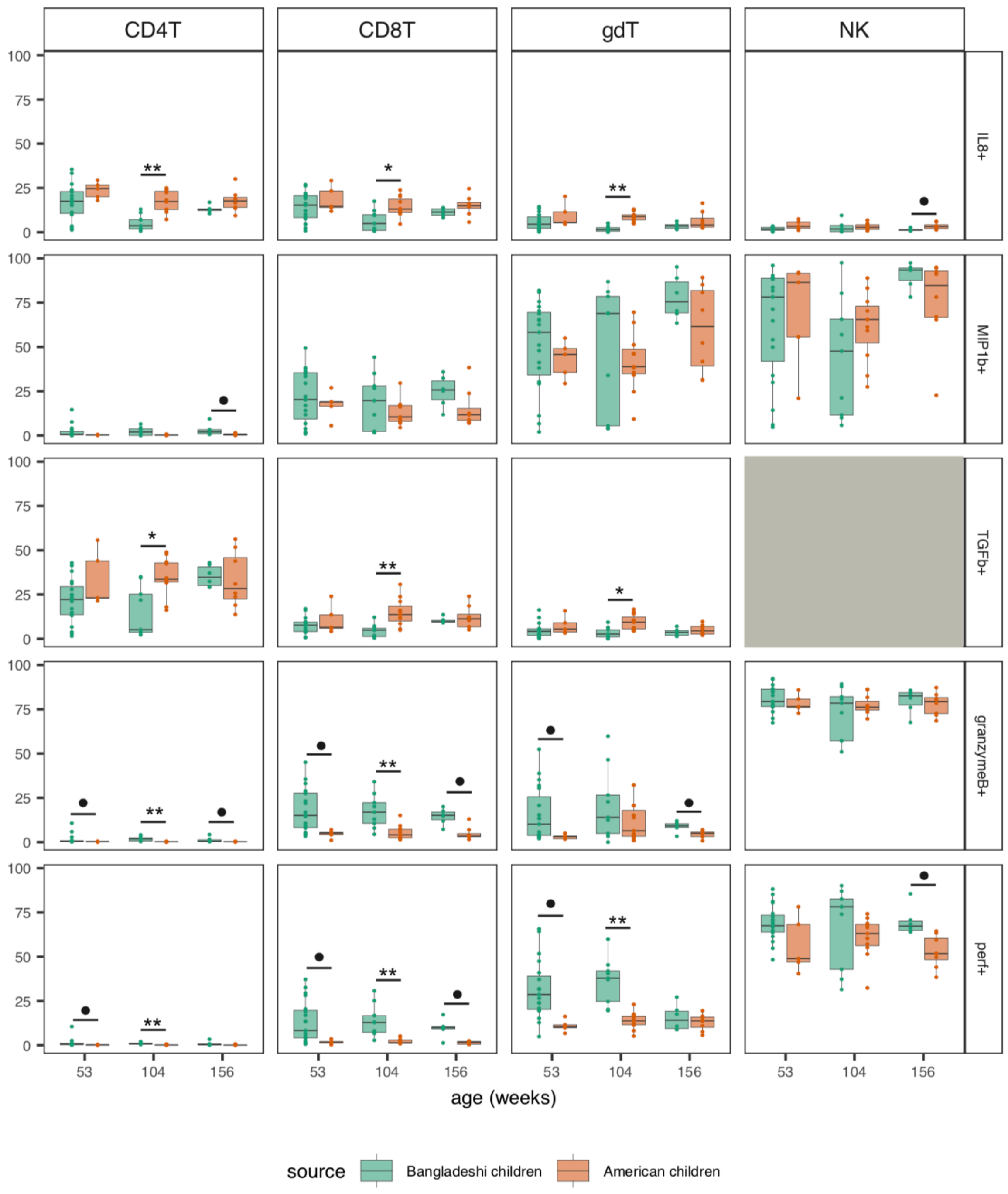

Supplementary Figure 3. Manual gating analysis of select cytokines from CD4 T, CD8 T, gamma delta T, and NK cell populations after PMA-ionomycin stimulation. To compare Bangladeshi and American children's immune cell populations from manual gating, Mann-Whitney tests were performed for each population and time point shown. *p* values were then corrected for multiple testing using the Benjamini & Hochberg method. Significant differences in cell frequencies between Bangladeshi and American children's samples are indicated as FDR<0.1 (•), FDR<0.05 (*), FDR<0.01 (**), FDR<0.001(***). Values plotted are means +/- SEM.

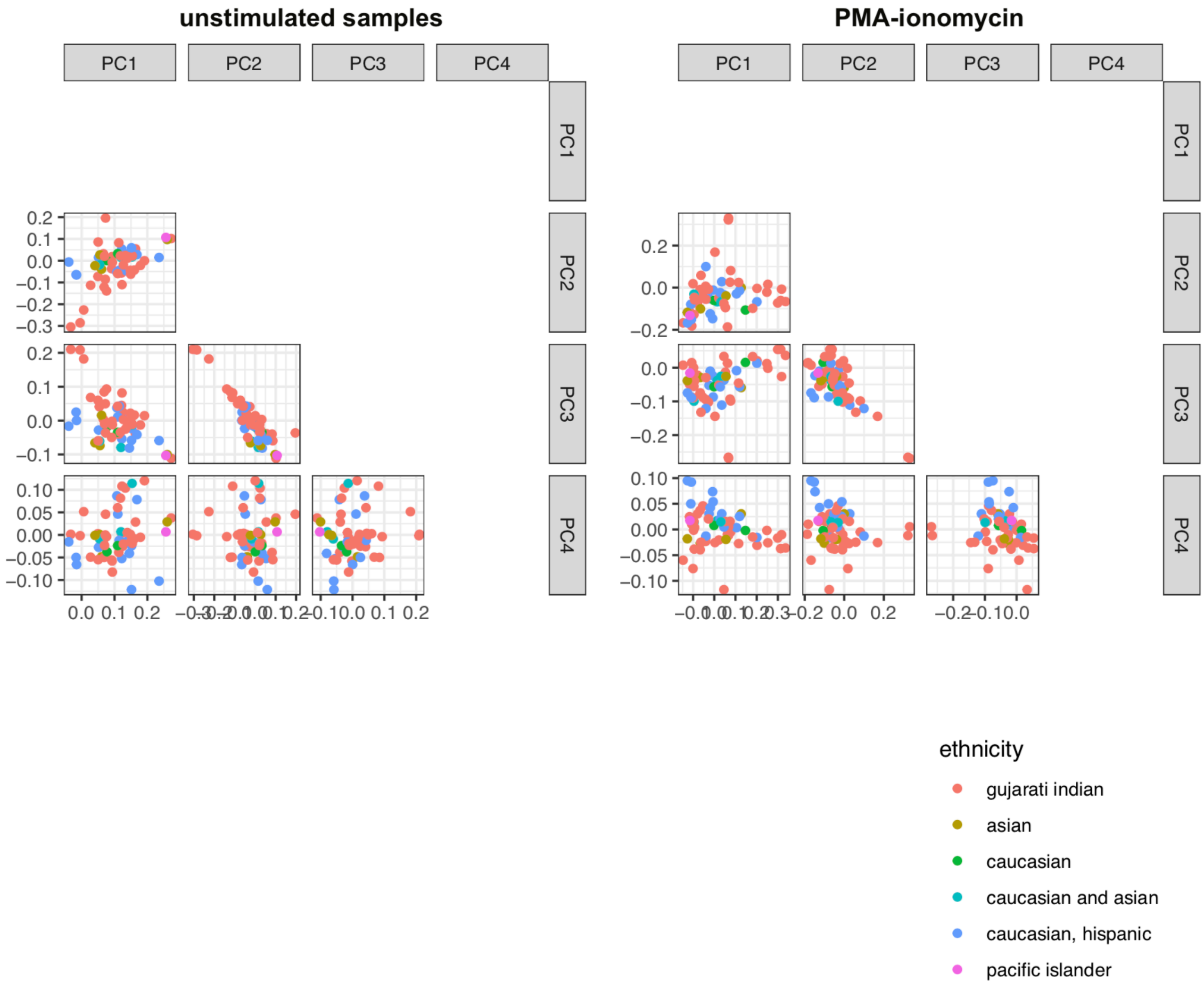

Supplementary Figure 4. Principal component analysis on AdjClust clustering groups from unstimulated and PMA-ionomycin stimulated samples. The first four components are shown plotted against one another for each stimulation condition with sample colors assigned based on ethnicity. All donors with ethnicity “Gujarati Indian” come from the Bangladeshi cohort.

Supplementary Table 1. Manually assigned definitions for clusters generated with AdjClust. Cluster assignments were made for **(A)** unstimulated and **(B)** PMA-ionomycin stimulated cluster maps based on marker heatmap expression and examination of individual marker intensities plotted as overlays on tSNE maps.

(A)

cluster	stimulation	definition	cellType
1	none	CD57+ CD4T	CD4 T cells
2	none	EMRA gdT	gd T cells
3	none	naive CD4T	CD4 T cells
4	none	EMRA gdT	gd T cells
5	none	CD57+ perf+ NK	NK cells
6	none	EMRA CD8+ gdT	gd T cells
7	none	CD57+ perf+ NK	NK cells
8	none	EMRA CD8T	CD8 T cells
9	none	perf+ NK	NK cells
10	none	NK	NK cells
11	none	EMRA CD8T	CD8 T cells
12	none	naive CD8T	CD8 T cells
13	none	naive CD8T	CD8 T cells
14	none	EMRA gdT	gd T cells
15	none	naive B	B cells
16	none	naive Treg	CD4 T cells
17	none	naive B	B cells
18	none	memory B	B cells
19	none	CD27+ memory CD4T	CD4 T cells
20	none	memory Treg	CD4 T cells
21	none	GzB+ CD8T	CD8 T cells
22	none	CD27- memory CD4T	CD4 T cells
23	none	CD56+ CD8T	CD8 T cells
24	none	memory CD8T	CD8 T cells
25	none	naive B	B cells
26	none	monocytes	monocytes
27	none	monocytes	monocytes
28	none	monocytes	monocytes

(B)

cluster	stimulation	definition	cellType
1	PMA-iono	non-activated CD4T	CD4 T cells
2	PMA-iono	non-activated CD8T	CD8 T cells
3	PMA-iono	activated CD8T	CD8 T cells
4	PMA-iono	CD40L+ CD4T	CD4 T cells
5	PMA-iono	CD40L+ CD4T	CD4 T cells
6	PMA-iono	non-activated Treg	CD4 T cells
7	PMA-iono	IL8+ CD8T	CD8 T cells
8	PMA-iono	CD40L+IL8+ CD4T	CD4 T cells
9	PMA-iono	IL8+ MIP1b+ mono	monocytes
10	PMA-iono	MIP1b+ CD8T	CD8 T cells
11	PMA-iono	MIP1b+GzB+perf+TNF+ gdT	gd T cells
12	PMA-iono	TGFb+ Treg	CD4 T cells
13	PMA-iono	activated B	B cells
14	PMA-iono	non-activated gdT	gd T cells
15	PMA-iono	activated B	B cells
16	PMA-iono	MIP1b+GzB+perf+TNF+ CD4T	CD4 T cells
17	PMA-iono	MIP1b+GzB+perf+TNF+ gdT	gd T cells
18	PMA-iono	IL2+CD40L+TGFb+ CD4T	CD4 T cells
19	PMA-iono	non-activated CD8T	CD8 T cells
20	PMA-iono	IL2+MIP1b+ CD8T	CD8 T cells
21	PMA-iono	IL2+CD40L+TGFb+ CD4T	CD4 T cells
22	PMA-iono	MIP1b+ NK	NK cells
23	PMA-iono	IL2+MIP1b+TNF+ CD8T	CD8 T cells
24	PMA-iono	activated gdT	gd T cells
25	PMA-iono	IL8+ B	B cells
26	PMA-iono	MIP1b+Gzb+perf+TNF+ CD8T	CD8 T cells
27	PMA-iono	MIP1b+GzB+perf+ NK	NK cells
28	PMA-iono	non-activated B	B cells
29	PMA-iono	IL8+ MIP1b+ mono	monocytes
30	PMA-iono	MIP1b+GzB+perf+ NK	NK cells

Supplementary Table 2. CyTOF staining panel and reagent sources.

label	polymer	marker	clone name	clone source	surface stain	IC stain
104Pd	SCN-Bn-EDTA	CD45	HI30	Biolegend	x	
106Pd	SCN-Bn-EDTA	CD45	HI30	Biolegend	x	
108Pd	SCN-Bn-EDTA	CD45	HI30	Biolegend	x	
113In	DN3	CD45	HI30	Biolegend	x	
115In	X8	CD57	HCD57	Biolegend	x	
139La	X8	CD16	3G8	Biolegend	x	
141Pr	X8	IL-8	BH0814	Biolegend		x
142Nd	X8	CD45RO	UCHL1	Biolegend	x	
143Nd	X8	perforin	B-D48	Abcam		x
144Nd	X8	IL-4	MP4-25D2	Fluidigm		x
145Nd	X8	CD33	WM53	Biolegend	x	
145Nd	X8	CD14	M5E2	Biolegend	x	
146Nd	X8	TNF	MAb11	Fluidigm		x
147Sm	X8	CD20	2H7	Biolegend	x	
148Nd	X8	IL-17a	BL168	Fluidigm		x
149Sm	X8	CD25	2A3	Fluidigm	x	
150Nd	DN3	MIP-1β	D21-1351	BD		x
151Eu	DN3	Granzyme B	GB11	BD		x
152Sm	DN3	CD103	B-Ly7	eBioscience	x	
153Eu	DN3	CD107a	H4A3	BD	during stimulation	
153Eu	DN3	CD107b	H4B4	BD	during stimulation	
154Sm	DN3	CD27	LG.7F9	eBioscience	x	
155Gd	X8	CD56	NCAM16.2	BD	x	
156Gd	X8	CD3	UCHT1	Biolegend	x	
157Gd	DN3	CD19	HIB19	Biolegend	x	
158Gd	X8	CD45RA	HI100	Biolegend	x	
159Tb	X8	CXCR5	RF8B2	BD	x	
160Gd	DN3	CD28	CD28.2	Biolegend	x	
161Dy	X8	CD38	HIT2	Biolegend	x	
162Dy	X8	CD69	FN50	Fluidigm		x
163Dy	X8	TGFβ1 (LAP)	TW4-6H10	Biolegend		x
164Dy	X8	IL-2	MQ1-17H12	eBioscience		x
165Ho	X8	IFNg	B27	Fluidigm		x
166Er	X8	IL-10	JES3-9D7	Fluidigm		x
167Er	X8	Integrin β7	FIB504	Biolegend	x	
168Er	DN3	CCR7	G043H7	Biolegend	x	
169Tm	X8	CD8	SK1	Biolegend	x	
169Tm	X8	IgD	IA6-2	Biolegend	x	
170Er	X8	CD4	SK3	Biolegend	x	
170Er	X8	IgA	G18-1	BD	x	
171Yb	X8	HLA-DR	L243	Biolegend	x	
172Yb	X8	LRP1	545503	R&D		x
173Yb	X8	TCRγδ	5A6.E9	in-house	x	
173Yb	X8	IgM	G20-127	BD	x	
174Yb	X8	CD40L	24-31	Biolegend		x
175Lu	X8	PD-1	EH12.2H7	Fluidigm	x	
176Yb	X8	CD127	A019D5	Biolegend	x	
191Ir	N/A	DNA1	-	Fluidigm		x
193Ir	N/A	DNA2	-	Fluidigm		x
195Pt	N/A	cisplatin	-	Fluidigm	x	

Supplementary Table 3. Markers used for tSNE preparation and relative weightings assigned for AdjClust clustering. For both unstimulated and PMA-ionomycin stimulated clustering, the minimum cluster size was set to 1,500 cells.

marker	stimulation	weight
CCR7	no stim	100
CD127	no stim	70
CD16	no stim	90
CD19	no stim	100
CD20	no stim	100
CD25	no stim	130
CD27	no stim	130
CD3	no stim	100
CD33+CD14	no stim	100
CD38	no stim	50
CD45RA	no stim	110
CD45RO	no stim	150
CD56	no stim	100
CD57	no stim	100
granzyme B	no stim	100
HLA-DR	no stim	90
IgA+CD4	no stim	90
IgD+CD8	no stim	110
IgM_gdTCR	no stim	120
perforin	no stim	100

marker	stimulation	weight
CCR7	PMA-iono	10
CD107a+CD107b	PMA-iono	40
CD127	PMA-iono	0
CD16	PMA-iono	20
CD19	PMA-iono	100
CD20	PMA-iono	100
CD25	PMA-iono	120
CD27	PMA-iono	130
CD3	PMA-iono	120
CD33+CD14	PMA-iono	100
CD38	PMA-iono	10
CD40L	PMA-iono	90
CD45RA	PMA-iono	120
CD45RO	PMA-iono	100
CD56	PMA-iono	100
CD57	PMA-iono	100
CD69	PMA-iono	130
granzyme B	PMA-iono	110
HLA-DR	PMA-iono	80
IFNg	PMA-iono	130
IgA+CD4	PMA-iono	120
IgD+CD8	PMA-iono	130
IgM+gdTCR	PMA-iono	90
IL-2	PMA-iono	130
IL-8	PMA-iono	130
MIP-1b	PMA-iono	110
perforin	PMA-iono	100
TGFb	PMA-iono	60
TNF	PMA-iono	110
